# What Can We Learn from the Evolution of Protein-Ligand Interactions to Aid the Design of New Therapeutics?

**DOI:** 10.1371/journal.pone.0051742

**Published:** 2012-12-11

**Authors:** Alicia P. Higueruelo, Adrian Schreyer, G. Richard J Bickerton, Tom L. Blundell, Will R. Pitt

**Affiliations:** 1 Department of Biochemistry, University of Cambridge, Cambridge, United Kingdom; 2 UCB Pharma, Slough, United Kingdom; Indian Institute of Science, India

## Abstract

Efforts to increase affinity in the design of new therapeutic molecules have tended to lead to greater lipophilicity, a factor that is generally agreed to be contributing to the low success rate of new drug candidates. Our aim is to provide a structural perspective to the study of lipophilic efficiency and to compare molecular interactions created over evolutionary time with those designed by humans. We show that natural complexes typically engage in more polar contacts than synthetic molecules bound to proteins. The synthetic molecules also have a higher proportion of unmatched heteroatoms at the interface than the natural sets. These observations suggest that there are lessons to be learnt from Nature, which could help us to improve the characteristics of man-made molecules. In particular, it is possible to increase the density of polar contacts without increasing lipophilicity and this is best achieved early in discovery while molecules remain relatively small.

## Introduction

The decrease in productivity in drug discovery and development, as measured by the cost of bringing a new medical entity (NME) to the market, is a many fold problem [1]. One of the main reasons for this well-documented decline is the poor physical properties of drug candidates entering into clinical trials ([2]–[5] and the references therein). Current leads are far too lipophilic to have good chances of success as safe drugs, as logP (calculated octanol-water partition coefficient) correlates positively with *in vitro* compound promiscuity [2]. For oral drugs, lipophilicity is generally considered a requirement for their absorption by passive diffusion in membranes, although there is some debate regarding the contribution of carrier-mediated transport to cellular uptake [6], [7]. Standard medicinal chemistry practices, driven by a “perceived need for potency” [8] and a sense of urgency to deliver leads into development, also contribute. In practical terms this has led to more lipophilic candidates, as the exploration of more risky molecules is discouraged [3]. This realization has led to the concept of lipophilic efficiency [2].

Nevertheless, trends in target selection are now providing further challenges. Although the focus on enzyme superfamilies such as protein kinases [9] and aspartic proteinases [10] has allowed knowledge and expertise to be transferred from one target to the next superfamily member, selectivity has proved more elusive than hoped. This has led to increasing interest in targeting protein-protein interactions [11], where the varied regulatory mechanisms within superfamilies promise a new avenue to selective drugs [12]. However, successful small molecule inhibitors of multi-protein complexes tend to be larger lipophilic molecules with few polar features [13], representing challenging starting points for the development of safe drugs. Here we ask whether this size and lipophilicity is a requirement that small molecules need to fulfill in order to bind to protein interfaces or merely a reflection on the nature of chemical matter that has been explored to date.

The aim of this analysis is to understand how Nature effects interactions and to migrate this knowledge to the design of small molecule modulators of biological targets. Although molecular recognition laws are far from simple, one can elucidate general trends in terms of atomic interactions from experimentally determined structures of natural protein complexes (not only multi-protein complexes but also endogenous small-molecule protein complexes) and compare them with trends from synthetic small molecule protein complexes. We therefore define interaction profiles in terms of polar and apolar contacts, with the aim of learning from natural patterns and incorporating them into the design of new therapeutics.

## Methods

### Compound set definition

Description and examples for each molecular set studied here can be found in File S1. PDB codes and redundancy flags can be downloaded from http://www-cryst.bioc.cam.ac.uk/members/alicia.

### Contact definition

Software to calculate hydrogen bonds for all types of molecules (proteins, nucleic acids and small molecules), with the same level of specificity, is not available at the moment. For this reason, simple polar and apolar contacts were defined using a simple distance cut-off of 4.5 Å. Contacts are labeled depending on the atom types of the proximal pair as follows:

#### Protein-protein complexes

Apolar contacts: C…C, C…S, S…S (not in Cys-Cys bridges).

Polar contacts: N…O, O…O, N…N, O…S, N…S (S from Cys).

#### Protein-small molecules complexes

Apolar contacts: C…C, C…S, C…X, S…X (X  =  Cl, Br, I).

Polar contacts: N…O, O…O, N…N, O…S, N…S, N…F, O…F, S…F (S from Cys).

This discrete count of atomic interactions correlates with buried surface area, which is used in other related studies. See File S2.

### Calculation of molecular properties

Molecular properties have been calculated using Pipeline Pilot (http://accelrys.com/products/pipeline-pilot/). The partition coefficient used in this study is AlogP. Rotatable bonds are defined as single bonds between heavy atoms. This does not include ring bonds (regardless of ring size) or those bonds that connect two heavy atoms, one of which is attached to only hydrogens. Furthermore, amide bonds are not considered to be rotatable.

### Statistical analysis

Distribution of polar/apolar contact ratio (normalized as polar/[polar+apolar]) between sets has been analyzed with non-parametrical tests (Kruskal-Wallis test for comparison of medians), as not all the sets have a normal distribution of the contact ratio.

## Results

### Data sets

A non-redundant set of protein-ligand complexes was extracted from the CREDO database [14] and classified into six sets by the type of small molecule involved: approved drugs, oral drugs, synthetic small molecules (for want of a better term, see File S1), protein-protein interactions inhibitors, natural small molecules and small peptides. These terms are used to describe the categories in the remaining sections of this paper. Five non-redundant sets of protein-protein complexes were extracted from the PICCOLO database [15] and classified as obligate dimers, transient dimers, homo and hetero pairwise interfaces from quaternary assemblies, and protein complexes successfully inhibited by small molecules. See File S1. PDB [16] codes for each subset are available to download at: http://www-cryst.bioc.cam.ac.uk/members/alicia.

### Polar and apolar contacts with protein molecules

Comparisons between different sets of molecules are based upon the number of polar and apolar atomic contacts. This discrete count of atomic interactions is analogous to the measurement of buried surface area used in other studies, providing a coarse description of the binding interfaces (see File S2).

**Figure 1:**
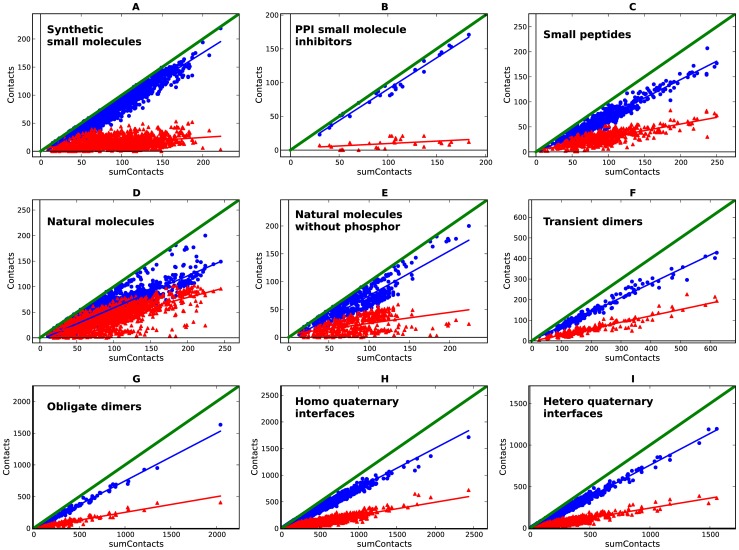
Scissors plots for the non-redundant-by-complex (Table S2) sets of protein complexes. a: synthetic small molecules bound to proteins; b: protein-protein interactions small molecule inhibitors bound to proteins; c: small peptides bound to proteins; d: natural small molecules bound to proteins; e: natural small molecules not containing phosphorus bound to proteins; f: transient protein-protein dimers; g: obligate protein-protein dimers; h: homo protein-protein interfaces from quaternary structures; i: hetero protein-protein interfaces from quaternary structures; polar (red) and apolar (blue) contacts are scattered against sum of contacts.

For each set of interfaces, plotting the total number of contacts (as sum of contacts) versus the number of both polar and apolar contacts generates the ‘scissors plot’. In such scissors plots, the relationship between the number of contacts and the polar and apolar contributions can be shown to be linear, with the gradient varying depending of the interaction type. The angle between the trend lines reflects the ratio of polar versus apolar contributions. Figure 1 shows the scissors plots for synthetic small molecules and PPI inhibitors (scissors open), natural small molecules (scissors closed) and small peptides and protein complexes (scissors half way). For the synthetic small molecules, the larger the interface, the greater the degree to which molecular interactions are dominated by apolar contacts, whereas the polar contacts remain relatively constant. The same conclusion was reached by Olsson and co-workers in their analysis of the SCORPIO database [17]. This observation is more pronounced in small molecules inhibiting protein-protein interactions as shown previously [13]. Conversely, natural small molecules, small peptides and protein complexes follow a different trend, where the polar interactions proportionally make a greater contribution. Interestingly, the lower left quadrant of the graphs, where the smaller ligands (fragments) are mostly located, shows a more balanced ratio between polar and apolar contacts. See File S3 for a different representation of these data.

This result agrees with the Hann complexity model [18], which maintains that it is easier for a smaller molecule to match target features, and also it supports the strategy of fragment-based drug design where the initial fragments anchor in a site with specific interactions [19] and where the step-by-step “growing” of the molecules, often guided by structure, tends to deliver less lipophilic hits [3],[5]. Natural molecules have a bimodal distribution as shown in Figure 1 (d and e) and Figure S1 (a); this is due to the presence of a subpopulation of steroid-like molecules, which have an apolar profile, amongst the population as a whole which tend to have more polar interactions. To evaluate whether this relatively high proportion of polar contacts is because many of the natural molecules have phosphate groups, Figure 1e shows the scissors plot for the set of natural molecules, which do not contain phosphorus. Despite the smaller sample size the same trend is observed, and the bimodal distribution is maintained. See File S4 for statistical significance of these plots.

Comparison of Figure 1a with Figure 1d and Figure 1e illustrates that natural molecules have a higher proportion of polar contacts than their synthetic counterparts. Likewise on average small peptides also engage in more polar contacts with their targets than synthetic molecules (Figure 1c versus 1a). In order to understand the determinants of these observed differences, the chemical structures of the different ligand sets were analyzed in terms of heteroatom content, rotatable bonds and the proportion of matched and unmatched atoms at the interfaces. The results of these analyses are shown below.

### Atomic composition and molecular flexibility

The heteroatom content of each molecule was measured by calculating the ratio of the number of non-carbon atoms to the total number of heavy (i.e. non hydrogen) atoms. The flexibility of each molecule was estimated by dividing the number of rotatable bonds by the number of heavy atoms. The average values for these metrics were then calculated for each of our sets, see File S5. The more polar interaction profile presented by natural molecules compared to synthetic small molecules coincides with a higher content of heteroatoms (26% on average in synthetic small molecules raises to 45% on average in natural molecules). Whereas peptides have a lower content of heteroatoms compared to natural molecules (36% on average). Peptides are also more flexible, the proportion of rotatable bonds by heavy atom is on average 18%, 22% and 42% for synthetic, natural molecules and small peptides respectively. This flexibility should make them more able to match the more directionally constrained polar interactions. In contrast to the natural molecules and peptide sets, the predominately apolar nature of the interactions for synthetic small molecules coincides with a lower heteroatom content and greater rigidity. However, examples exist of natural molecules that are rigid and lipophilic, including steroids such as testosterone. In fact, natural molecules cover a broad range of polarity, but the overall character is predominantly polar, especially when compared with synthetic molecules.

### Matched and unmatched atoms at the binding interfaces

As we have shown, the higher content of heteroatoms in non-peptidic natural molecules often leads to more polar interactions with the protein. It is also clear from our analyses that this is not the case for small peptides but the lower heteroatom content may be compensated for by their greater flexibility, allowing them to form more polar interactions. However, it is important to understand what proportion of heteroatoms in each class of molecule is making polar contacts or is unmatched (i.e. heteroatoms within the 4.5 Å threshold that do not satisfy any polar criteria). In other words, what are the polar contact efficiencies?

**Figure 2:**
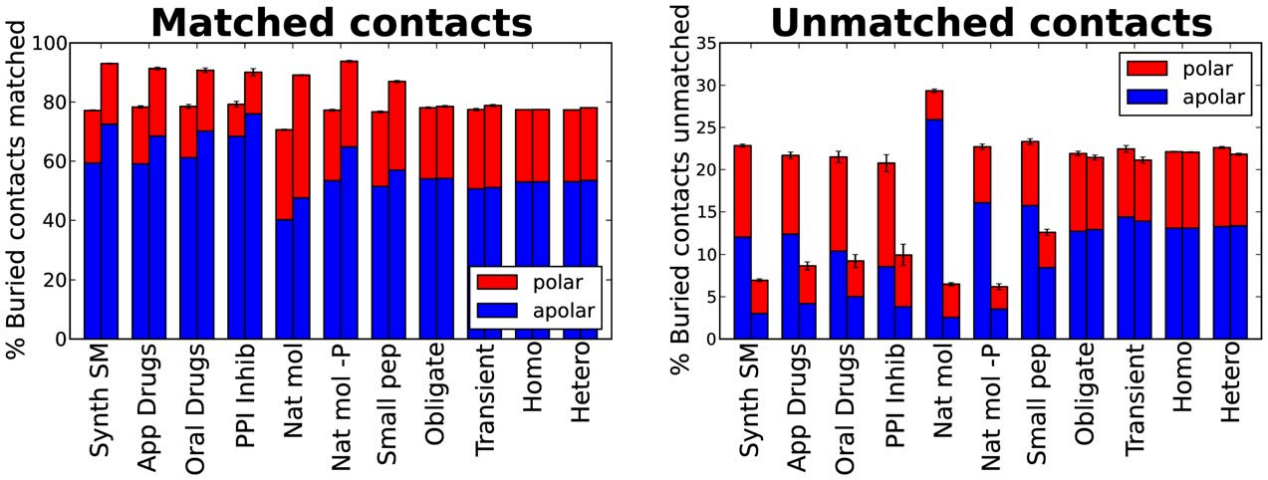
Matched and unmatched contacts. Mean of the percentage of buried atoms per molecule engaged in successful interactions (Matched contacts, left chart) and mean of the percentage of buried atoms without an appropriate partner in the other side of the interface (Unmatched contacts, right chart). The percentage is divided into polar (red) and apolar (blue) contribution. Each set has two bars, one on the left for the atoms in the protein and one on the right for the atoms in the ligand or smaller protein in the case of protein complexes. Error bars denote the standard error of the mean. Sets are ordered from left to right: Synthetic small molecules, approved drugs, oral drugs, protein-protein interaction small molecule inhibitors, natural molecules, natural molecules without phosphor, small peptides, obligate protein-protein dimers, transient protein-protein dimers, homo quaternary protein-protein interfaces and hetero quaternary protein-protein interfaces.

Figure 2 shows the means of the proportions of matched and unmatched buried atoms. Small molecule ligands, which are represented on the left hand side of the figure, are more contact efficient than the protein to which they are bound. On average around 90% of the ligand atoms are matched in all sets, whereas only 70–80% of the protein atoms are matched. Natural molecules without phosphorus are the most contact efficient, 70–80% of the protein atoms are matched. The small molecule atoms are more exposed and able to contact the protein, whereas the atoms in the protein can be less accessible probably due to the concave nature of ligand binding pockets. Furthermore, studies of ligand-binding pockets have shown that they are on average one and a half to three times bigger than the ligand they encapsulate [20], [21] (the exact ratio varies depending on the dataset studied and the algorithm used). Therefore, in proportion more atoms in the protein will be at the periphery of the ligand (our cut-off here was 4.5 Å) without making useful interactions. Another interesting result from this analysis is that synthetic molecules have a higher proportion of unmatched polar atoms (on both ligand and protein side) than the natural ones. Therefore, if one wants to increase the polar contacts synthetic molecules make, then there is opportunity for improvement. Oral drugs need to have a limited polar surface area in order to be absorbed by the body [22]. However, Figure 2 shows that approved and oral drugs are not making the most of their polar composition. Nevertheless, improving enthalpic contacts is not a trivial task, not only because of the difficulty of designing chemical geometries that match polar constraints, but also because of the increased desolvation penalty and the reduction of conformational entropy [23] that can result.

**Figure 3:**
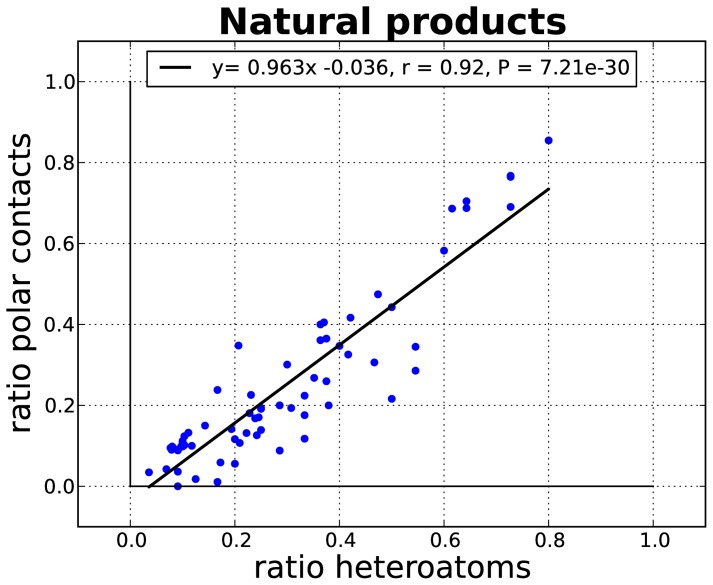
Linear regression of the proportion of heteroatoms to the polar ratio (as number of polar contacts divided by the total [polar + apolar] number of contacts) for the natural-product-like subset of molecules.

The question remains, does Nature make the most of the polar composition of its ligands? Figure 3 shows a linear correlation between heteroatom content (the number heteroatoms divided by the total number of atoms) and the ratio of polar interactions (the number polar contacts divided by the sum of contacts, henceforth referred to as the polar ratio), for the natural-product-like subset in natural molecules. For these small molecules, the increase in polar features translates into more polar interactions with the protein.

### Synthetic small molecule complexes. Calculated properties versus interaction profile

**Figure 4:**
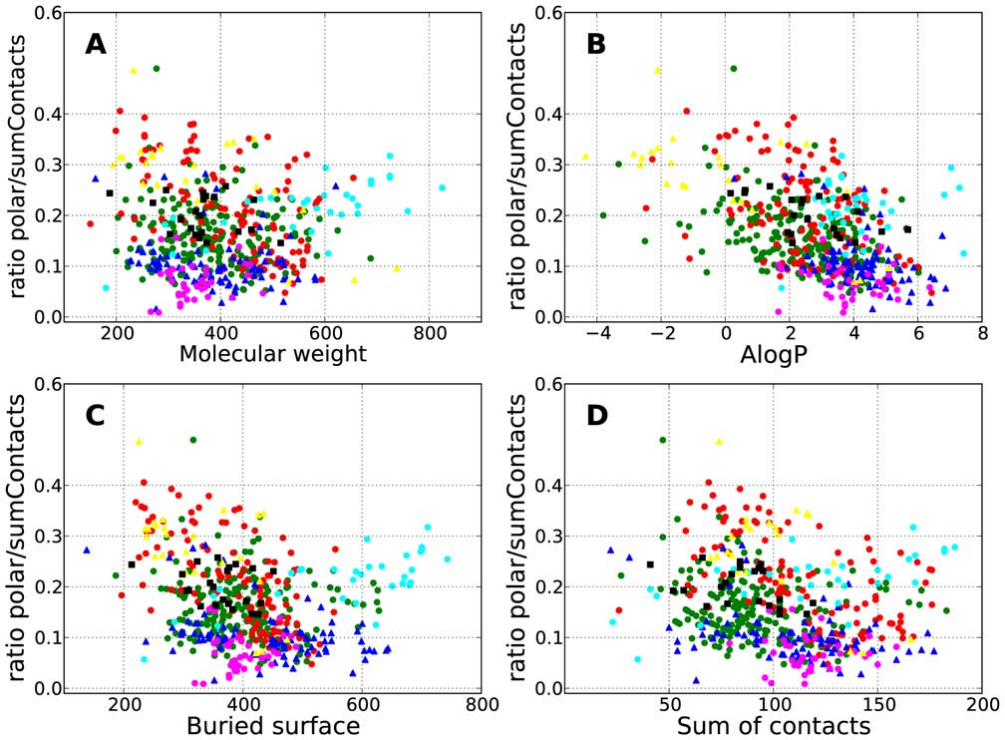
Calculated properties versus interaction profile. The polar ratio (as number of polar contacts divided by the total [polar + apolar] number of contacts) versus molecular weight (A), AlogP (B), buried area upon binding (C) and sum of contacts (D) for protein complexes with synthetic small molecules. Different colors denote SCOP families: Protein kinase catalytic subunit (green), nuclear receptor ligand-binding domain (blue), eukaryotic proteases (red), retroviral proteases – retropepsin (cyan), reverse transcriptase (magenta), Higher-molecular weight phosphotyrosine protein phosphatases (yellow), HSP90 N-terminal domain (black). For clarity, only SCOP families binding to more than 20 different ligands are shown.

An analysis of the distribution of the polar ratio plotted against molecular weight, AlogP, surface area buried upon binding and sum of contacts has been carried out for the synthetic small molecules. Figure 4 shows these distributions color-coded by SCOP [24] family.

Synthetic molecules bound to protein kinases (green dots in Figure 4) tend to have a high AlogP and a low polar ratio (usually below 30%). Even those molecules with a negative AlogP do not appear to make many polar contacts. However, there are a few molecules with 50% of polar contacts, for example HET-ID 3C3 in PDB entry 2CGW. In the case of nuclear receptor ligand-binding domain (NR-LBD, blue triangles in Figure 4), all the molecules have AlogP>1 and most have less than 15% of polar contacts, but as with the kinase ligands there are examples that buck the trend, for example HET-ID 444 in PDB entry 1UHL with a polar ratio of 34%). Eukaryotic proteases (red dots in Figure 4) bind to a wide range of molecules with molecular weights ranging from 200 to 700 Da and with AlogPs between −2 and 6. They also have a wide range of polar ratios. Bigger and more lipophilic molecules are found to bind to retroviral proteases (cyan dots in Figure 4) with similar polar binding patterns as found for eukaryotic enzymes, although polar fragments are also found. The proteins belonging to the reverse transcriptase SCOP family have similar characteristics to nuclear receptor ligand-binding domain, and bind to molecules with AlogP>1, all of which have less than 15% polar contacts. Most of the molecules bound to phosphotyrosine protein phosphatases (PTPP, yellow triangles in Figure 4) have around 30% of polar contacts with a low AlogP range (−2 to 2), although there are also three apolar binders (HET-ID 892 in PDB entry 1T49, HET-ID BB3 in PDB entry 1T48 and HET-ID FRJ in PDB entry 1T4J). However, these apolar molecules are inhibitors binding to an allosteric site. Finally, HSP90 domains (black squares in Figure 4) bind to molecules with a wide range of AlogP (0–6) with between 15–25% of polar contacts.

The characteristics of synthetic small molecules depend heavily upon their targets. However, there is no correlation between calculated lipophilicity or molecular weight and the polar ratio. Thus, it is possible to achieve more polar contacts without compromising the physical properties of the drug leads. See Table S1 for a list of approved and oral drugs with more than 40% of polar contacts. The low lipophilicity of these molecules is noteworthy. In a recent analysis of patented small molecules from 18 pharmaceutical companies, Leeson and St-Gallay [5] show that here is indeed a target dependency of the lead lipophilicity. However, the outcomes of different companies working on the same target also highlight that the major determinant of the lipophilicity of the compounds is the discovery practices of each organization and not target requirements.

### Synthetic small molecule complexes. Affinity versus interaction profile

Affinity data from the implementation of PDBBind [25] in CREDO [14] are available for almost 700 of the synthetic small molecule set and more than one hundred of the small peptides. For these molecules there is no correlation between the binding energy and the proportion of polar contacts they make with their protein partners (File S6). Small, weakly binding synthetic molecules have high polar ratios, whereas small peptides can engage more polar contacts across a wide range of affinities.

**Figure 5:**
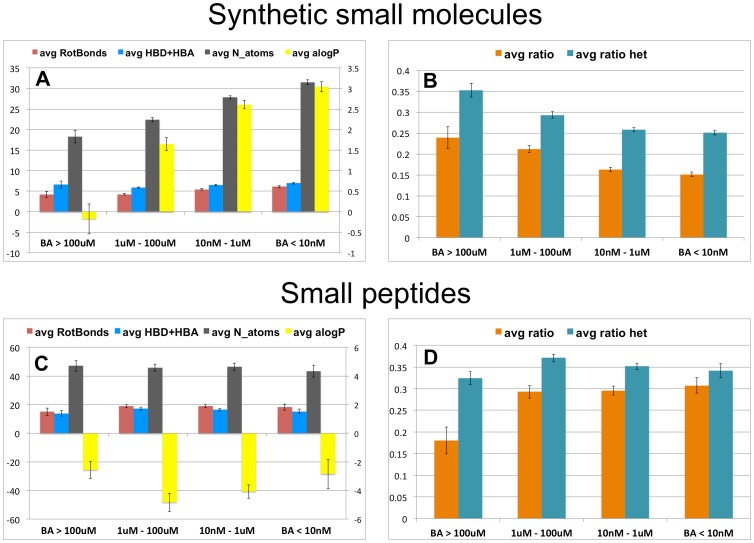
Binding affinity versus interaction profile. Binned binding affinity (BA) data for synthetic small molecules (A and B) and for small peptides (C and D). Bars in (A) and (C) denote the average of molecular properties for each affinity bin: rotatable bonds (red), sum of hydrogen bond donors and acceptors (blue), number of atoms (black) and AlogP (yellow, in the secondary right axis scale for clarity). Bars in (B) and (D) denote the average of polar ratio (orange) and the average heteroatom content (cyan). Error bars are the standard error of the mean.

Indeed, Figure 5a shows that the most potent synthetic molecules have the highest average number of atoms and AlogP while the average count of hydrogen bond acceptors and donors remains constant across the whole potency range. This result is in agreement with the much discussed general tendency in drug discovery of gaining potency by adding lipophilic groups to the small molecules; see for example [2]. In the set studied here, this translates (Fig. 5b) into lower heteroatom content and a lower polar ratio.

Figure 5 (c and d) shows that small peptides have on average the same property profile regardless of their potency. This result suggests that small peptides do not achieve tight binding through increase of lipophilicity. Furthermore the proportion of polar contacts and the heteroatom content is maintained across the whole range of affinities with the exception of weak binders where the polar ratio is lower (although there are only seven complexes in this category). The important point to highlight here is that peptides, unlike synthetic small molecules, can bind specifically to their receptors and have a high number of polar contacts. We argue that this is in part due to their inherent flexibility and low lipophilicity. Note that the average number of rotatable bonds in small peptides is four-fold higher than in synthetic molecules. In fact, the small peptides studied here are often bigger than the synthetic small molecules, as no size limit was applied to select the small peptide set, whereas synthetic molecules larger than 900 Da were omitted. However, there is no correlation between the number of atoms and the free energy of binding for small peptides, see File S7, in agreement with the conclusions previously reached by Kuntz [26]. Nevertheless, they are less efficient than small synthetic molecules as they use more atoms to achieve the same affinity.

### Small molecule inhibitors of protein-protein interactions

We now return to the question of whether small molecules inhibiting protein-protein interactions need to be large and lipophilic. Although these molecules tend to be lipophilic with few polar features, Figure 2 shows that they have polar atoms unmatched in the binding site. Using the TIMBAL database [13], we have extracted the seven cases where there is structural information for both the small molecule-protein and the protein-protein complexes. In all cases studied, there are unutilized potential polar contacts on the protein interface (see Table 1 and File S8 for a graphical representation of these data). These cases illustrate a common pattern for synthetic molecules: they typically comprise few anchor points (which can be considered as more constrained polar contacts) and more potency-boosting hydrophobic interactions. Small molecule inhibitors of protein-protein interactions do not take advantage of all the available opportunities for polar contacts in the interfaces as only a few of them are engaged. For these seven cases, comparison of the interacting residues in the target protein highlights that small molecules tend to use more aromatic and fewer charged residues than the protein partner, see Figure S2.

**Table 1:** Examples of polar ratio in protein complexes.

Target	PDB p-p	Ratio p-p	Affinity	PDB p-sm	Ratio p-sm	Affinity	refs
**IL-2**	1Z92 (A:B)	**0.35**	10nM (Kd)	1PY2 (A)	**0.21**	60nM (IC50)	[27],[28]
**Bcl-XL**	2BZW (A:B)	**0.19**	6nM (Kd)	2YXJ (B)	**0.08**	0.6nM	[29]
**MDM2**	1YCR (A:B)	**0.14**	600nM (Kd)	1T4E (A)	**0.03**	67nM (Kd)	[30],[31]
**XIAP**	1G3F (A:B)	**0.22**		1TFT (A)	**0.12**		[32], [33]
**ZipA**	1F47 (B:A)	**0.10**	21.6uM (Kd)	1Y2F (A)	**0.00**	12uM (Kd)	[34],[35]
**TNF**	1TNF (AB:C)	**0.30**	-	2AZ5 (C&D)	**0.12**	13uM	[36], [37]
**S100B**	1DT7 (A:X)	**0.34**	-	3GK1 (A)	**0.12**		[38],[39]

Polar ratio is the number of polar contacts divided by the total [polar + apolar] number of contacts. These protein complexes are for proteins that bind to both protein partners (ratio p-p, left) and synthetic small molecules (ratio p-sm, right). The PDB code includes the interacting chains, for example 1TNF(AB:C) denotes chain A and B interacting with chain C of the TNF trimer, whereas 2AZ5 (C&D) denotes chains C and D interacting with the small molecule. When available, affinity measure and units is specified in table.

## Discussion

In this study we have analyzed the atomic contacts between different sets of molecules, some natural and some synthetic. The results presented here show that natural complexes typically engage in more polar contacts than synthetic molecules bound to proteins. This latter set, also have a higher proportion of unmatched heteroatoms than the natural sets and, probably for this reason, show no correlation between lipophilicity and proportion of polar contacts. These differences may be due to the fact that the dynamics of evolutionary processes are more likely to provide a more highly optimized configuration than is possible by medicinal chemistry efforts. Importantly it must be borne in mind that endogenous molecules have not been constrained by the requirements for oral absorption and distribution across membranes into cellular compartments.

For synthetic small molecules in general, but in particular for the inhibitors of protein-protein interactions, we conclude that efforts should be invested to maximize polar contacts to better resemble the interaction patterns that natural molecules present. As the ratio of polar versus apolar contacts is greater when the size of the synthetic molecules is smaller, this will be easiest to achieve when ligands remain small. However, it will almost always be harder to match polar contacts than to gain affinity through the addition of hydrophobic substituents. Nevertheless, the recent analysis of Leeson and St-Gallay [5] shows that for the same target, a range of compound lipophilicity can be achieved using different strategies, thus encouraging efforts that may lead to better molecules. If we are to minimize ligand promiscuity and ultimately lower the costs of delivering safe and effective drugs to the market, it must be worthwhile to “go back to Nature” and see what can be learnt from the exquisite specificity – albeit low ligand efficiency – of protein-ligand interactions that has evolved to be selectively advantageous in living organisms.

## Supporting Information

Figure S1 Normalized distributions of the ratio of polar contacts (represented by polar/[polar+apolar]), each chart compares synthetic small molecules against the other sets.
(PDF)

Figure S2 Comparisons of residue propensities at the binding sites for small molecule protein-protein inhibitors versus protein-protein complexes inhibited by them.
(PDF)

File S1 Detailed description of each data set.
(PDF)

File S2 Buried surface area versus atomic contacts.
(PDF)

File S3 More polar contacts for synthetic fragments.
(PDF)

File S4 Comparison of interaction profiles.
(PDF)

File S5 Heteroatom content and rotatable bond count.
(PDF)

File S6 **Free energy of ligand binding versus the polar ratio of contacts [polar/(polar+apolar)] for the synthetic small molecules set and the small peptide set.**
(PDF)

File S7 **Free energy of ligand binding versus the number of atoms of the ligand for the synthetic small molecules set and the small peptide set.**
(PDF)

File S8 Protein-protein complexes inhibited by small molecules.
(PDF)

Table S1 Approved and oral small molecule drugs that engage more than 40% polar contacts with their bound protein.
(PDF)

Table S2 Number of entries in each set of molecules.
(PDF)
