# Supplementary material for: What Can We Learn from the Evolution of Protein-Ligand Interactions to Aid the Design of New Therapeutics?
Source: PLoS One. 2012 Dec 11;7(12):e51742. doi: 10.1371/journal.pone.0051742 (PMC3519888; doi:10.1371/journal.pone.0051742)
Supplement: Figure S1 — Normalized distributions of the ratio of polar contacts (represented by polar/[polar+apolar]), each chart compares synthetic small molecules against the other sets. (PDF) [file pone.0051742.s001.pdf]

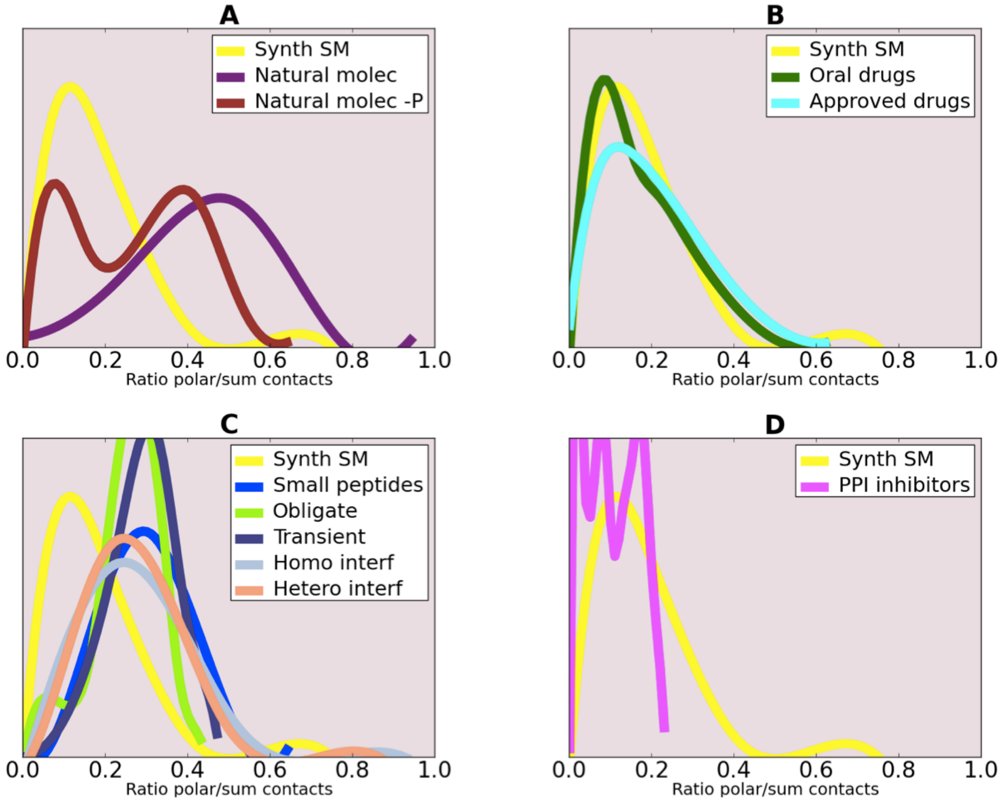

Supplementary Figure S1. Normalized distributions of the ratio of polar contacts (represented by polar/[polar+apolar]), each chart compares synthetic small molecules against the others. (A): synthetic versus natural small molecules with and without phosphor. (B): synthetic versus approved and oral drugs. (C): synthetic versus small peptides, obligate and transient protein-protein dimers, homo and hetero quaternary protein-protein interfaces. (D): synthetic versus PPI inhibitors.
