# Supplementary material for: What Can We Learn from the Evolution of Protein-Ligand Interactions to Aid the Design of New Therapeutics?
Source: PLoS One. 2012 Dec 11;7(12):e51742. doi: 10.1371/journal.pone.0051742 (PMC3519888; doi:10.1371/journal.pone.0051742)
Supplement: Figure S2 — Comparisons of residue propensities at the binding sites for small molecule protein-protein inhibitors versus protein-protein complexes inhibited by them. (PDF) [file pone.0051742.s002.pdf]

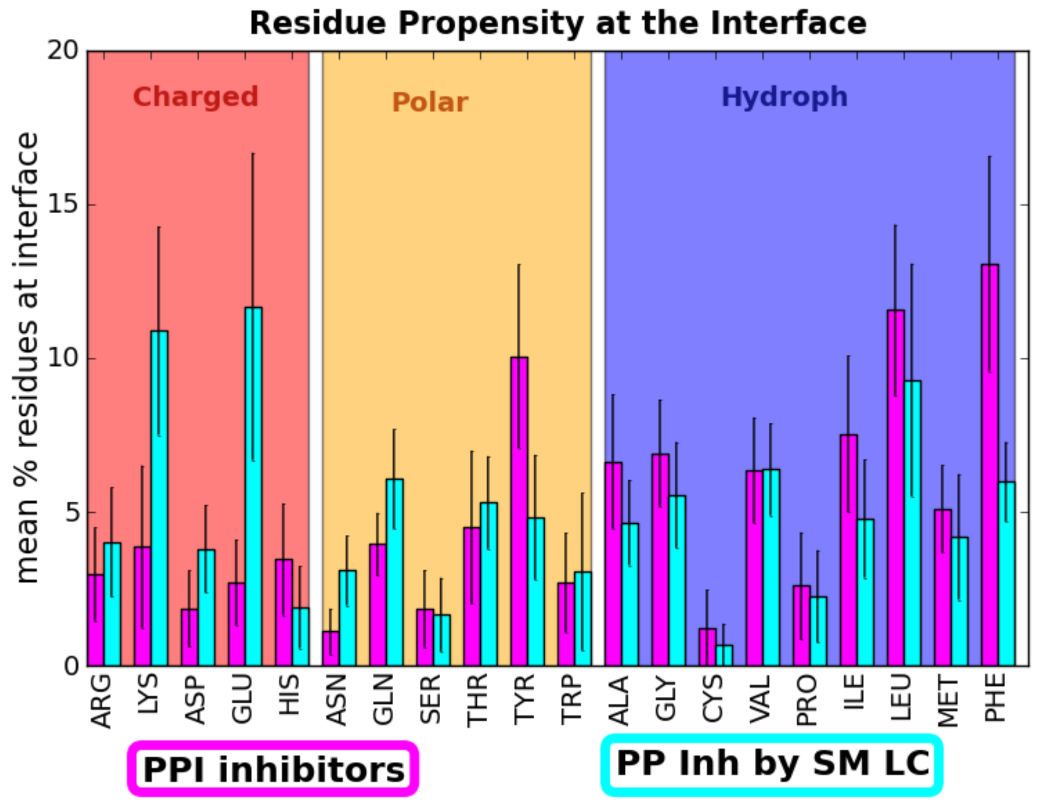

Supplementary Figure S2. Comparisons of residue propensities at the binding sites for small molecule protein-protein inhibitors (magenta) versus protein-protein complexes inhibited by them (cyan), note these subsets are small (9 and 7 complexes respectively). For protein-protein complexes only the long chain (LC) is considered. Bar heights represent the mean percentage of each residue at the interface. Error bars denote the standard error of the mean. The background color represents whether the residue is charged (red), polar (orange) or hydrophobic (blue).
