# Supplementary material for: What Can We Learn from the Evolution of Protein-Ligand Interactions to Aid the Design of New Therapeutics?
Source: PLoS One. 2012 Dec 11;7(12):e51742. doi: 10.1371/journal.pone.0051742 (PMC3519888; doi:10.1371/journal.pone.0051742)
Supplement: File S1 — Detailed description of each data set. (PDF) [file pone.0051742.s003.pdf]

What can we learn from the evolution of protein-ligand interactions to aid the design of new therapeutics?

Alicia P. Higueruelo[1], Adrian Schreyer[1], G. Richard J. Bickerton[1,2], Tom L. Blundell[1] and Will R. Pitt[1,3]

[1]Department of Biochemistry, University of Cambridge, Cambridge, UK

[2]Present address: Division of Biological Chemistry and Drug Discovery, College of Life Sciences, University of Dundee, Dundee, UK

[3]UCB Pharma, Slough, UK

Correspondence should be addressed to APH (alicia@cryst.bioc.cam.ac.uk)

Supplementary File S1

SF1.1 Data sets

Supplementary Table SF1.T1 summarizes the number of entries for each non-redundant set of protein-small molecule and protein-protein complexes. Some classifications inevitably exhibit a certain degree of ambiguity, for example the definition of "natural product" is inherently rather arbitrary. By the same argument, "drug-like molecule" classification is not unequivocal; it is more a continuous "likeness" property without rigorous thresholds. Furthermore, the emerging new targets have forced debate about what it takes to be a drug [1]. The molecular property thresholds applied to select "drug-like" molecules in this study are somewhat loose, for instance the molecular weight cut-off is 900. The reason for these broad filters is to be able to compare like to like with the small molecules inhibiting protein-protein complexes (molecular weight range: 150-815Da). Therefore, the term "synthetic small molecules" is used instead of drug-like across the manuscript. In other cases, the annotation seems to be accurate and straightforward but

misinterpretation occurs nevertheless. For example, the case of the "citrate anion", a common buffer to maintain neutral pH in experimental conditions and therefore a common ligand in the PDB [2]. Due to its size (13 atoms), this ligand can be easily labeled as an oral drug, as lithium citrate (or carbonate) is commonly used to treat depression. However, the active ingredient is the lithium, not the counter anion.

Set	Unique by Complex	Unique UniProt	Unique SCOP families
**Synthetic small molecules**	1,525 (1,206)	518 (385)	165 (143)
**Approved drugs**	201 (95)	155 (76)	67 (46)
**Oral drugs**	134 (68)	93 (49)	24 (19)
**Protein-protein interaction inhibitors**	30 (25)	9 (9)	7 (7)
**Natural molecules**	1505 (283)	1159 (216)	346 (134)
**Small peptides**	557 (467)	288 (238)	98 (83)
**Obligate dimers**	161	161	293
**Transient dimers**	154	154	183
**Homo quaternary interfaces**	12,034	7,177	2,711
**Hetero quaternary interfaces**	2,271	1,709	897
**Protein-protein complexes SM inhibited**	15	15	13

Supplementary Table SF1.T1. Number of entries in each set of molecules. The non-redundant sets are considering non-redundant set of interactions for the complexes (protein-ligand or protein-protein interaction). From these sets protein redundancy is removed by selecting unique UniProt [3] identifiers and removed structural domains redundancy by selecting unique SCOP [4] families. Numbers in parenthesis are the number of unique small molecules in each set. Numbers for unique UniProt and SCOP families for protein complexes refer to distinct pairs of UniProt identifiers or SCOP family respectively.

SF1.2 Small molecule protein-protein interaction inhibitors

Small molecule inhibitors of protein-protein complexes were identified using TIMBAL [5]. Visual inspection of the 39 PDB entries stored in TIMBAL yielded 28 non-redundant protein-small molecule complexes. Entries with non-biological contacts were removed, i.e. other than ligand-protein interactions, such as ligand-ligand. Supplementary Figure SF1.F1 shows examples of chemical structures from this set.

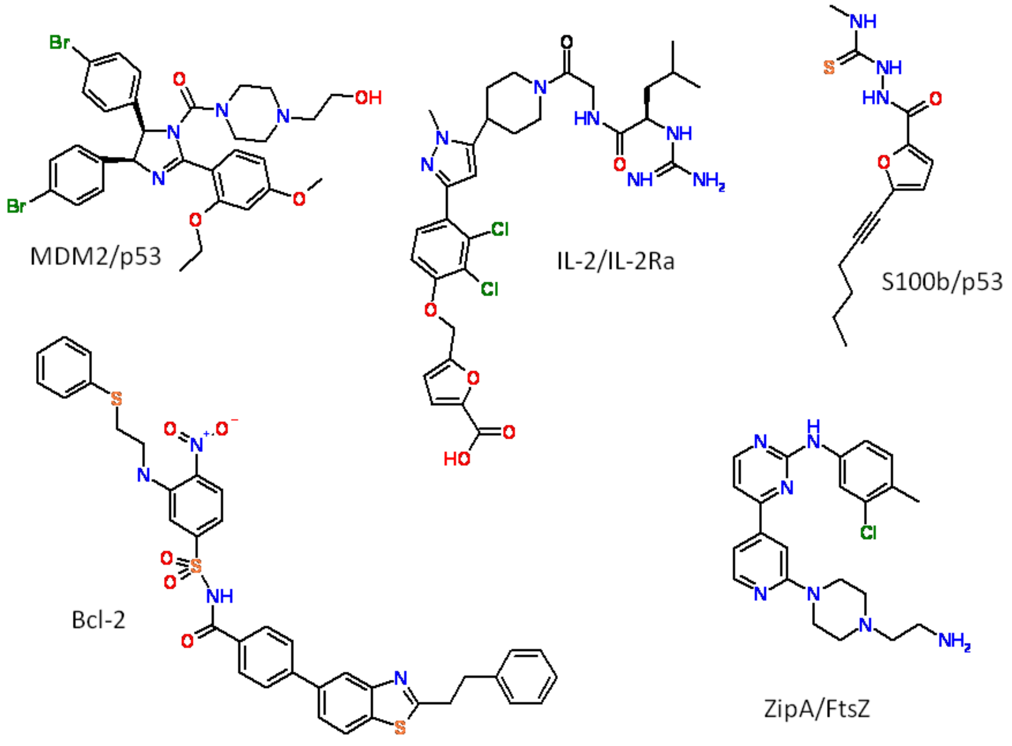

Supplementary Figure SF1.F1. Examples of chemical structures of the small molecules bound protein-protein complexes. Each structure is labeled with the protein complex it inhibits or binds to.

SF1.3 Natural molecules

Natural small molecules were identified with KEGG [6], HMDB [7], ChEMBL [8], MGEx (pure natural products from AnalytiCon Discovery, http://www.ac-discovery.com) databases implemented in CREDO. Natural small molecules in this set are:

- Ligands flagged as substrate, product or cofactor from KEGG
- Ligands labeled as endogenous from the HMDB
- Natural products from MGEx
- Ligands that are a least 90% similar to small molecules classified in ChEMBL as natural products

Filters and redundancy removal yielded 1,505 non-redundant complexes between natural small molecules and proteins, from which there were only 283 distinct small molecules. Supplementary Figure SF1.F2 shows that half of this non-redundant subset of interactions was composed of eight nucleotide small molecules: ADP, NAD, NAP, ATP, AMP, FAD, SAH and COA. This redundancy and the chemical composition are taken into account in the discussion. For example, all of these eight molecules have sugar rings and all but SAH (S-adenosyl-l-homocysteine) have phosphates, therefore these molecules have a high content in heteroatoms.

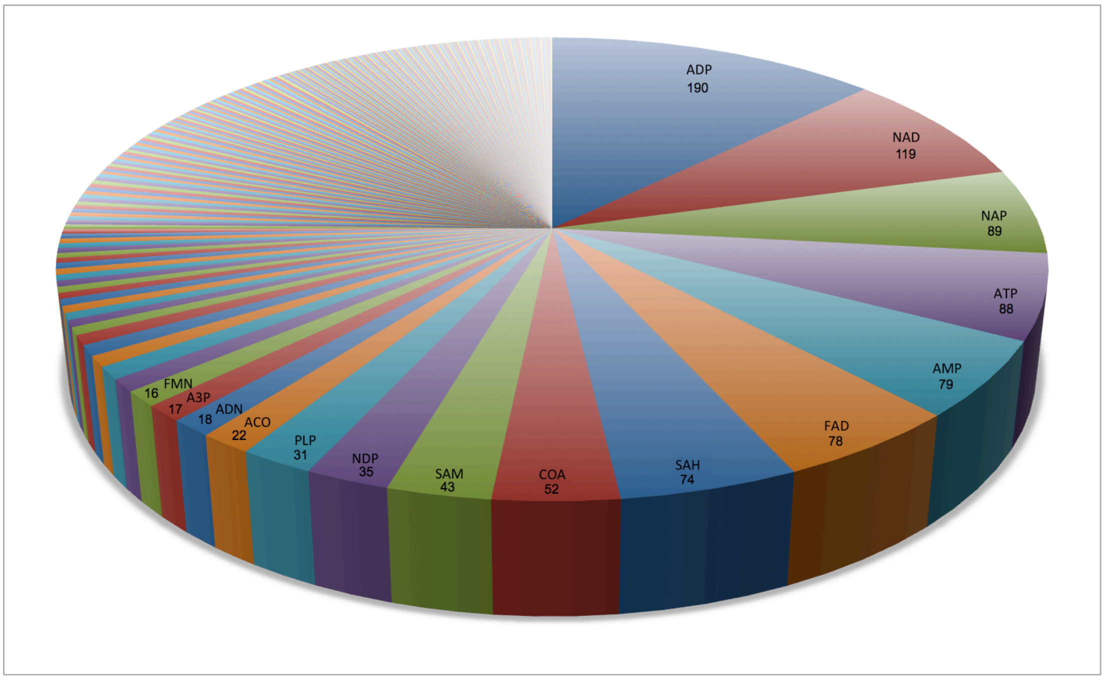

Supplementary Figure SF1.F2. Distribution of the natural small molecule subset in terms of entries per chemical structure of the small molecule bound to protein. Only higher frequency entries are labeled for clarity. Note more than half of the subset is composed of the complexes with eight different molecules: ADP, NAD, NAP, ATP, AMP, FAD, SAH and COA.

This diverse set of molecules was manually classified as antibiotics (13 chemical structures), lipids (13 chemical structures), natural-product-like (72 chemical structures), nucleotides (104 chemical structures), peptide-like (16 chemical structures), steroids (37 chemical structures) and sugars (28 chemical structures). Supplementary Figure SF1.F3 shows an example of chemical structures from each category for this set.

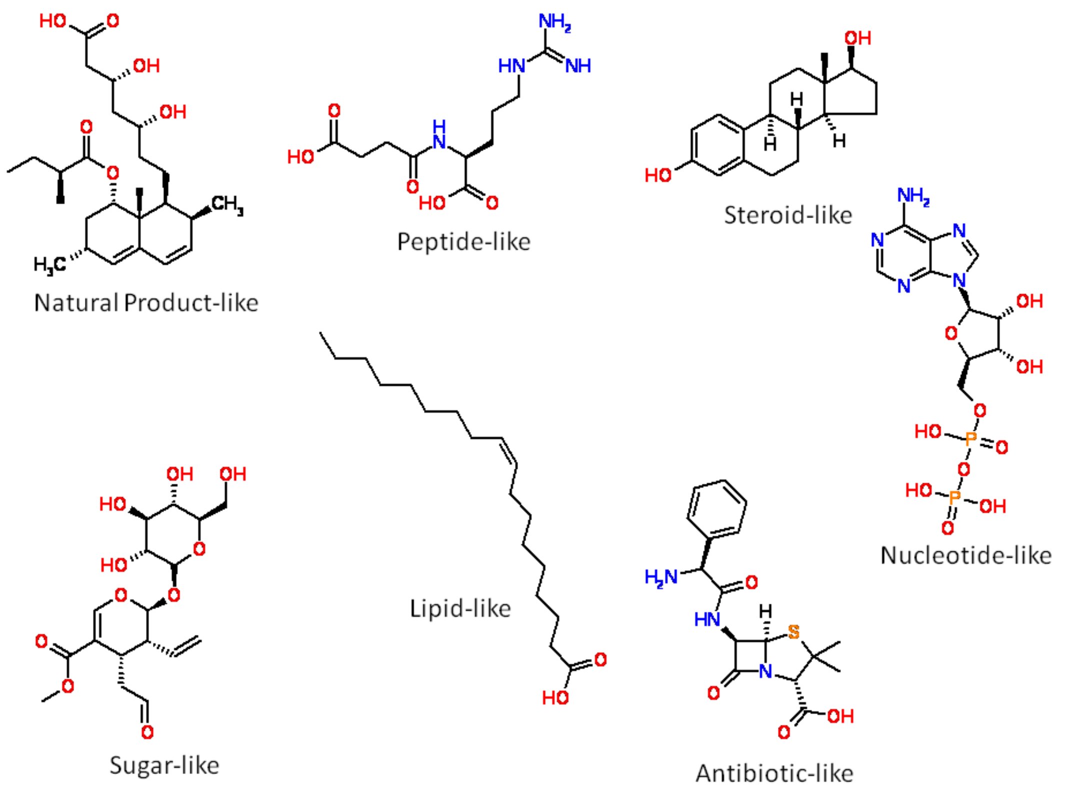

Supplementary Figure SF1.F3. Examples of chemical structures in the natural molecules set. Labels correspond to the manual classification based in their structures and functions, so these molecules are categorized into natural product like, peptide like, steroid like, sugar like, lipid like, antibiotic like and nucleotide like.

SF1.4 Small peptides

This subset includes short peptides of up to eight residues. These residues can be standard and non-standard amino acids as well as any other residue type, as long as at least half of them are standard amino acids. Examples of molecules in this subset can be seen in Supplementary Table SF1.T2.

PDB	Chain id	Residue list	Std_aa/ length
2IFR	B	ACE-PHE-LYS-PHE-TA2-ALA-LEU-ARG	6/8
1BZH	I	ASP-ALA-ASP-GLU-FLT-LEU-AEA	5/7 cyclic
2FNX	P	VAL-ILE-ALA-LYS	4/4
1CE1	P	GLY-THR-SER-SER-PRO-SER-ALA-ASP	8/8

Supplementary Table SF1.T2. Examples of ligands in the small peptide set. Last column refers to the ratio of number of standard amino acids by the total residue length of the ligand.

SF1.5 Synthetic small molecules

In order to avoid overlapping, molecules of this set have been selected from the PDB after extracting the small molecules from the previous sets. Therefore, this set comprises mainly synthetic man-made molecules. Molecules in this set have:

- At least one carbon atom and one ring, are composed only by carbon, nitrogen, oxygen, sulphur, halogen, chains no longer than six sp3-CH2 and less than 900Da of molecular weight

Molecules with the following features are excluded:

- Covalent or metal bonded with proteins
- Peptide-like molecules using an amino acid SMARTS filter [9]
- Recognized solvents (initial set from [10] and manually extended by Adrian Schreyer in CREDO) or having fewer than 10 atoms
- Belong to structures containing nucleic acids
- Similar to nucleotide analogues or detergents
- Have alternate locations for the ligands or residues in the interface
- Result from crystal structure analyses where the resolution is lower than 3.5Å
- Also exist in the small molecule inhibitors of protein-protein complexes, natural molecules or small peptides sets

Supplementary Figure SF1.F4 shows the chemical diversity of this set and the fine line between definitions, for example ligand MVB could be selected as natural molecule.

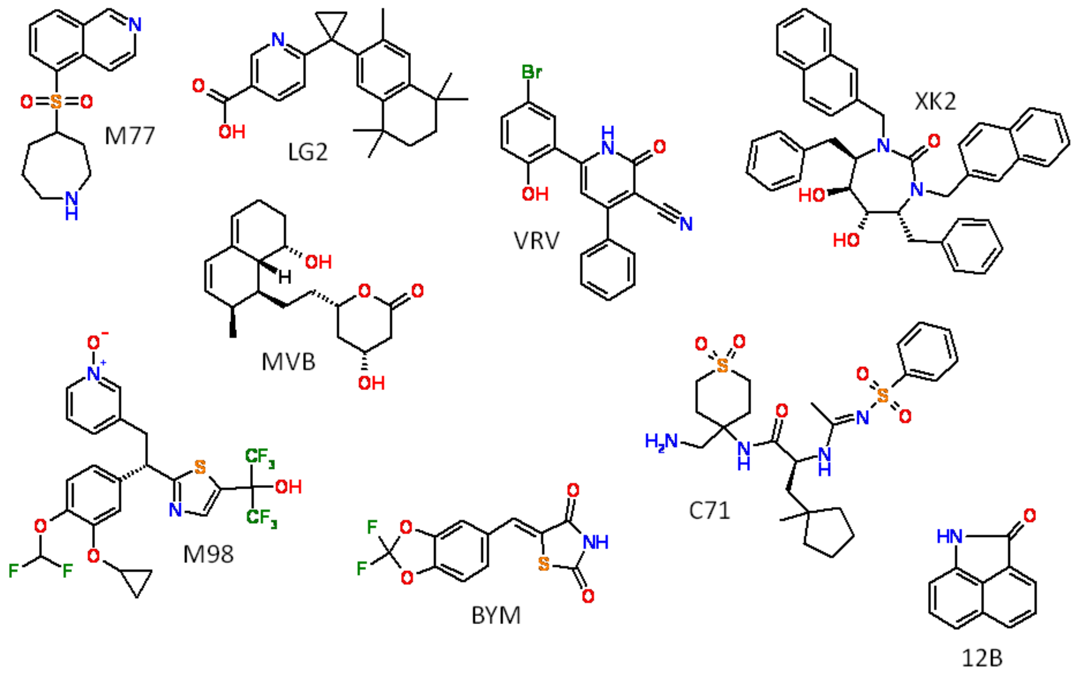

Supplementary Figure SF1.F4. Examples of chemical structures in the synthetic small molecule subset. Molecules are labeled with their hetID (residue) identifier from the PDB.

SF1.6 Approved and oral drugs

Molecules from this set were selected from the classification in DrugBank [11] as approved drugs and from the classification in ChEMBL [8] as oral drugs. However, molecules in the approved set can have any administration route, including oral. These molecules were manually classified as antibiotics (25 chemical structures), lipids (two chemical structures), natural-product-like (29 chemical structures), nucleotides (six chemical structures), peptide-like (10 chemical structures), steroids (14 chemical structures), sugars (six chemical structures) and NOTA (none of the above) (65 chemical structures). Supplementary Figure SF1.F5 shows an example of chemical structures from each category for this set. The complexes studied are not necessarily the ligand-drug with its intended target. For example, in PDB 2BXF Diazepam (Valium, positive allosteric modulator of $GABA_A$ receptor) is bound to human serum albumin. Furthermore, the "approved drug" label also comprises molecules like Thiamin (vitamin B1, example of natural-product-like in Supplementary Figure SF1.F5) or Ascorbic acid (vitamin C, example of sugar-like in Supplementary Figure SF1.F5). There are also cases of molecules that were marketed but were later withdrawn, for example Bextra (Valdecoxib, example of NOTA in Supplementary Figure SF1.F5). All these data are not easily accessible, either stored in a standardized manner, however molecules in this set were kept as models of small molecules that successfully made their way into the body with a therapeutic effect.

In terms of the size, molecules with fewer than 10 atoms have been removed from all sets. However, there are approved drugs of that size. For example, guanidine with four atoms is an approved oral treatment of myasthenia (DrugBank ID DB00536), or dimethyl sufoxide, also with four atoms, is a common solvent but also an

approved topical analgesic (DrugBank ID DB01093). Nevertheless, the filter of a minimum of 10 atoms has been maintained even for this set, as such small molecules are more common as additives in the experimental solutions than as biologically relevant entities.

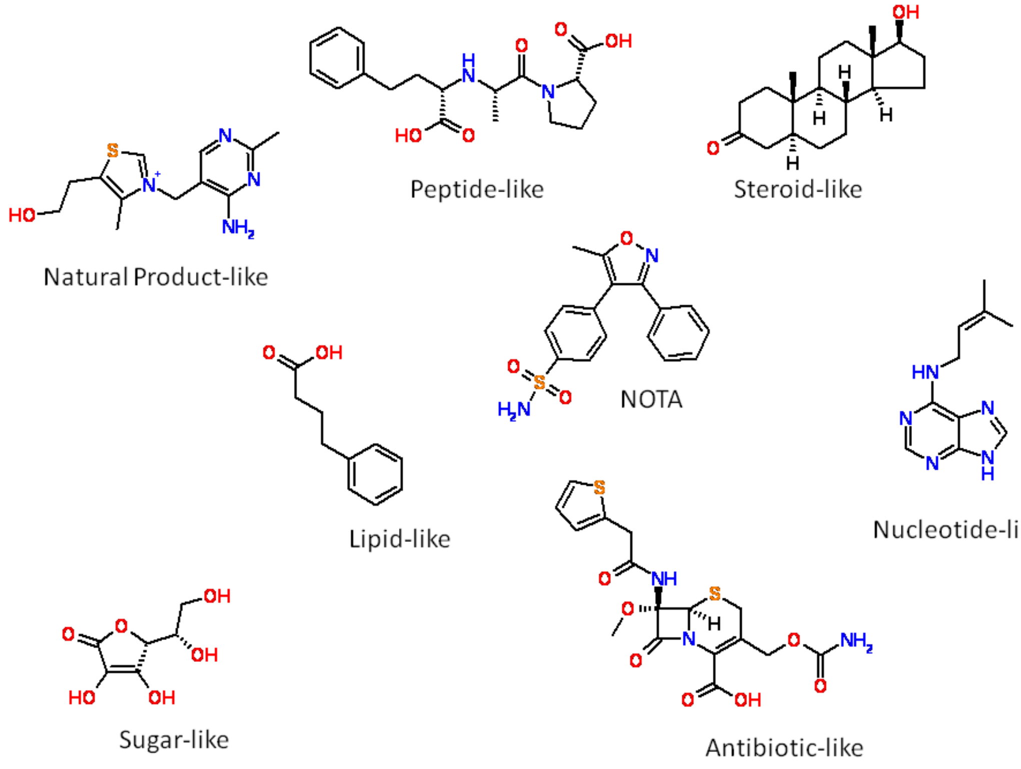

Supplementary Figure SF1.F5. Examples of chemical structures in the approved and oral drugs set. Labels correspond to the manual classification based on their structures, so these molecules are categorized into natural product like, peptide like, steroid like, sugar like, lipid like, antibiotic like, nucleotide like and none of the above (NOTA).

SF1.7 Protein-protein sets

No further classification has been done for the protein sets. In this study, only protein interfaces are considered regardless of their function, or which constituents form the complex, for example antigen-antibody, enzyme-inhibitor or protein-receptor. The only categorization used refers to the lifetime of the complexes: permanent (labeled as obligate) and transient dimers from the publicly available sets [12,13]. These were small sets (315 entries with both dimer classes), but were kept in order to capture any difference in binding pattern, such as transient complexes are more likely to be targeted by a small molecule drug. On the other hand, the general non-redundant set of protein-protein interfaces was considered from PICCOLO, from the quaternary structures predicted by PISA [14]. These interfaces were further divided into hetero- (different proteins interacting) and homo- (same protein interacting).

References

1. Macarron R, Luengo JI (2011) Yin and Yang in medicinal chemistry: what does drug-likeness mean? Future Medicinal Chemistry 3: 505-507.
2. Berman HM, Westbrook J, Feng Z, Gilliland G, Bhat TN, et al. (2000) The Protein Data Bank. Nucleic Acids Res 28: 235-242.
3. Apweiler R, Bairoch A, Wu CH, Barker WC, Boeckmann B, et al. (2004) UniProt: the Universal Protein knowledgebase. Nucleic Acids Res 32: D115-119.
4. Murzin A, Brenner S, Hubbard T, Chothia C (1995) SCOP: a structural classification of proteins database for the investigation of sequences and structures. J Mol Biol 247: 536–540.
5. Higueruelo AP, Schreyer A, Bickerton GRJ, Pitt WR, Groom CR, et al. (2009) Atomic Interactions and Profile of Small Molecules Disrupting Protein-Protein Interfaces: the TIMBAL Database. Chem Biol Drug Des 74: 457-467.

6. Kanehisa M, Goto S, Furumichi M, Tanabe M, Hirakawa M (2010) KEGG for representation and analysis of molecular networks involving diseases and drugs. Nucleic Acids Res 38: D355-360.
7. Wishart DS, Knox C, Guo AC, Eisner R, Young N, et al. (2009) HMDB: a knowledgebase for the human metabolome. Nucleic Acids Res 37: D603-610.
8. Gaulton A, Bellis LJ, Bento AP, Chambers J, Davies M, et al. (2011) ChEMBL: a large-scale bioactivity database for drug discovery. Nucleic acids research 40: D1100-D1107.
9. Daylight SMARTS query for aminoacids. [$([$([NX3H,NX4H2+]),$([NX3](C)(C)(C))]1[CX4H]([CH2][CH2][CH2]1)[CX3](=[OX1])[OX2H,OX1-,N]),$([$([NX3H2,NX4H3+]),$([NX3H](C)(C))][CX4H2][CX3](=[OX1])[OX2H,OX1-,N]),$([$([NX3H2,NX4H3+]),$([NX3H](C)(C))][CX4H]([*])[CX3](=[OX1])[OX2H,OX1-,N])].
10. Hartshorn MJ, Verdonk ML, Chessari G, Brewerton SC, Mooij WT, et al. (2007) Diverse, high-quality test set for the validation of protein-ligand docking performance. J Med Chem 50: 726-741.
11. Knox C, Law V, Jewison T, Liu P, Ly S, et al. (2011) DrugBank 3.0: a comprehensive resource for 'omics' research on drugs. Nucleic Acids Res 39: D1035-1041.
12. Zhu H, Domingues FS, Sommer I, Lengauer T (2006) NOXclass: prediction of protein-protein interaction types. BMC Bioinformatics 7: 27.
13. Mintseris J, Weng Z (2005) Structure, function, and evolution of transient and obligate protein-protein interactions. Proc Natl Acad Sci U S A 102: 10930-10935.
14. Krissinel E, Henrick K (2007) Inference of macromolecular assemblies from crystalline state. J Mol Biol 372: 774-797.
