# Supplementary material for: What Can We Learn from the Evolution of Protein-Ligand Interactions to Aid the Design of New Therapeutics?
Source: PLoS One. 2012 Dec 11;7(12):e51742. doi: 10.1371/journal.pone.0051742 (PMC3519888; doi:10.1371/journal.pone.0051742)
Supplement: File S2 — Buried surface area versus atomic contacts. (PDF) [file pone.0051742.s004.pdf]

What can we learn from the evolution of protein-ligand interactions to aid the design of new therapeutics?

Alicia P. Higueruelo[1], Adrian Schreyer[1], G. Richard J. Bickerton[1,2], Tom L. Blundell[1] and Will R. Pitt[1,3]

[1]Department of Biochemistry, University of Cambridge, Cambridge, UK

[2]Present address: Division of Biological Chemistry and Drug Discovery, College of Life Sciences, University of Dundee, Dundee, UK

[3]UCB Pharma, Slough, UK

Correspondence should be addressed to APH (alicia@cryst.bioc.cam.ac.uk)

Supplementary File 2

Buried surface versus atomic contacts

Olsson and co-workers [1] studied the binding between small molecules and proteins from the Scorpio database of Isothermal Titration Calorimetry (ITC) data in terms of polar and apolar proportion of buried surface area upon binding. Similarly, we have based comparisons between different sets of molecules on the extent of polar and apolar atomic contacts that they make. This discrete count of atomic interactions resembles the measurement of buried surface area used in other studies, providing a coarse description of interface. Supplementary Figure SF2.F1 shows the correlation of the buried surface area and the number of contacts for all the small molecules used in the analysis. Supplementary Table SF2.T1 shows correlation data for each subset and contact type. In all cases there was significant linear correlation between the surface area buried upon binding and the atomic contacts the

small molecule made with the protein. For all cases, r value was 0.8 which shows a medium-strong correlation between the data [2].

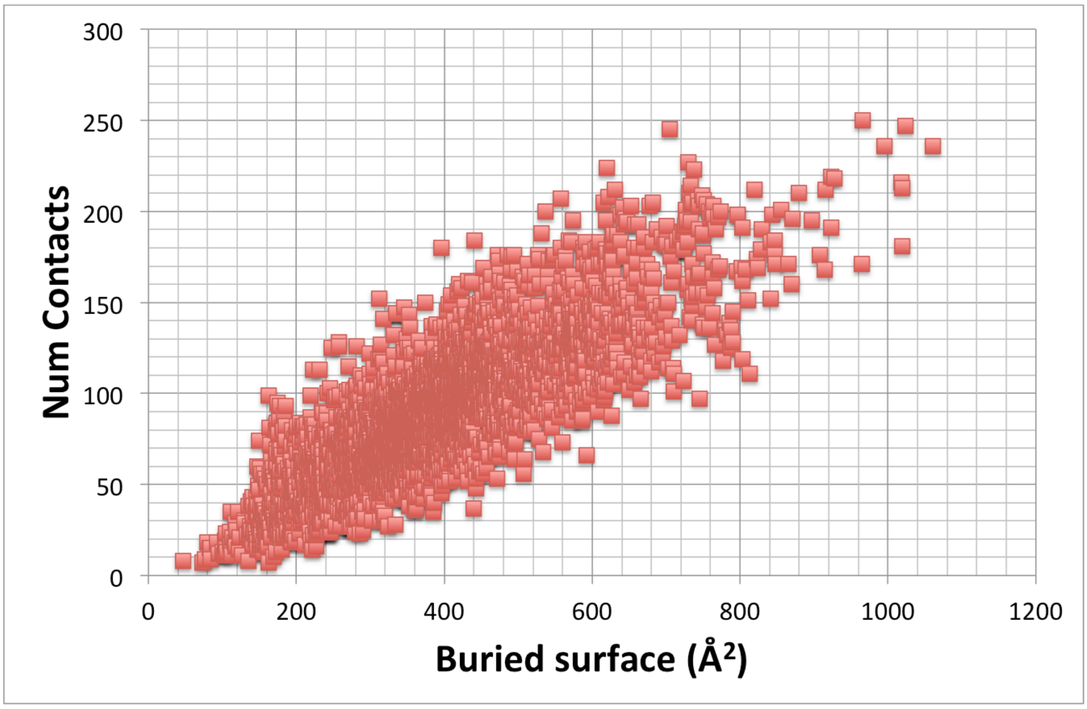

Supplementary Figure SF2.F1. Scatter plot of buried surface area upon binding and the number of atomic contacts (polar and apolar) the small molecules made. Points are from all small molecule sets: synthetic small molecules, approved drugs, oral drugs, protein-protein interaction inhibitors, natural molecules and small peptides.

Subset	Contact type	r value	P value
**Synthetic small molecules**	all	0.82	0.00
	polar	0.85	0.00
	apolar	0.82	0.00
**Approved drugs**	all	0.79	3.86E-45
	polar	0.83	4.36E-53
	apolar	0.76	4.11E-39
**Oral drugs**	all	0.89	1.04E-67
	polar	0.85	1.37E-56
	apolar	0.90	1.54E-73
**Protein-protein interaction inhibitors**	all	0.84	2.03E-08
	polar	0.89	1.52E-10
	apolar	0.81	1.34E-07
**Natural molecules**	all	0.85	0.00
	polar	0.91	0.00
	apolar	0.81	0.00
**Small peptides**	all	0.79	0.00
	polar	0.84	0.00
	apolar	0.73	8.77E-93
**All (SupFig 6)**	all	0.82	0.00
	polar	0.90	0.00
	apolar	0.82	0.00

Supplementary Table SF2.T1. r and P values from linear correlation calculations between buried surface upon binding and number of atomic contacts small molecules make with proteins. P value has been rounded to zero when P < 1E-100.

References

1. Olsson TSG, Williams MA, Pitt WR, Ladbury JE (2008) The Thermodynamics of Protein-Ligand Interaction and Solvation: Insights for Ligand Design. J Mol Biol 384: 1002-1017.
2. Townend J (2002) Correlation and regression. Practical Statistics for Environmental and Biological Scientists. reprint 2007 ed: John Wiley & Sons Ltd. pp. 129-152.
