# Supplementary material for: What Can We Learn from the Evolution of Protein-Ligand Interactions to Aid the Design of New Therapeutics?
Source: PLoS One. 2012 Dec 11;7(12):e51742. doi: 10.1371/journal.pone.0051742 (PMC3519888; doi:10.1371/journal.pone.0051742)
Supplement: File S3 — More polar contacts for synthetic fragments. (PDF) [file pone.0051742.s005.pdf]

What can we learn from the evolution of protein-ligand interactions to aid the design of new therapeutics?

Alicia P. Higueruelo[1], Adrian Schreyer[1], G. Richard J. Bickerton[1,2], Tom L. Blundell[1] and Will R. Pitt[1,3]

[1]Department of Biochemistry, University of Cambridge, Cambridge, UK

[2]Present address: Division of Biological Chemistry and Drug Discovery, College of Life Sciences, University of Dundee, Dundee, UK

[3]UCB Pharma, Slough, UK

Correspondence should be addressed to APH (alicia@cryst.bioc.cam.ac.uk)

Supplementary File 3

More polar contacts for synthetic fragments

Supplementary Figure SF3.F1 shows the ratio of polar versus sum of contacts binned by sum of contacts. With this representation, it becomes clear that the molecules engaging more polar contacts, for example in the synthetic small molecules set, have fewer contacts overall and they are generally smaller molecules. This effect is more pronounced in the small molecule inhibitors of protein interfaces. A similar situation occurs with natural molecules without phosphorus, the polar proportion of contacts decreases with ligand size. This becomes more evident in Supplementary Figure SF3.F2, where the upper right quadrant of the nine charts is empty. In these graphs (Supplementary Figure SF3.F2), the proportion of polar contacts (Y axis) decreases with molecular size (X axis as sum of contacts). This result can be justified in terms of the Hann's complexity model [1], the chances of matching at the same time different polar interactions decreases with the number of interactions to match.

Furthermore, the flexibility required to match many different specific interactions goes against spontaneous binding due to entropic penalty.

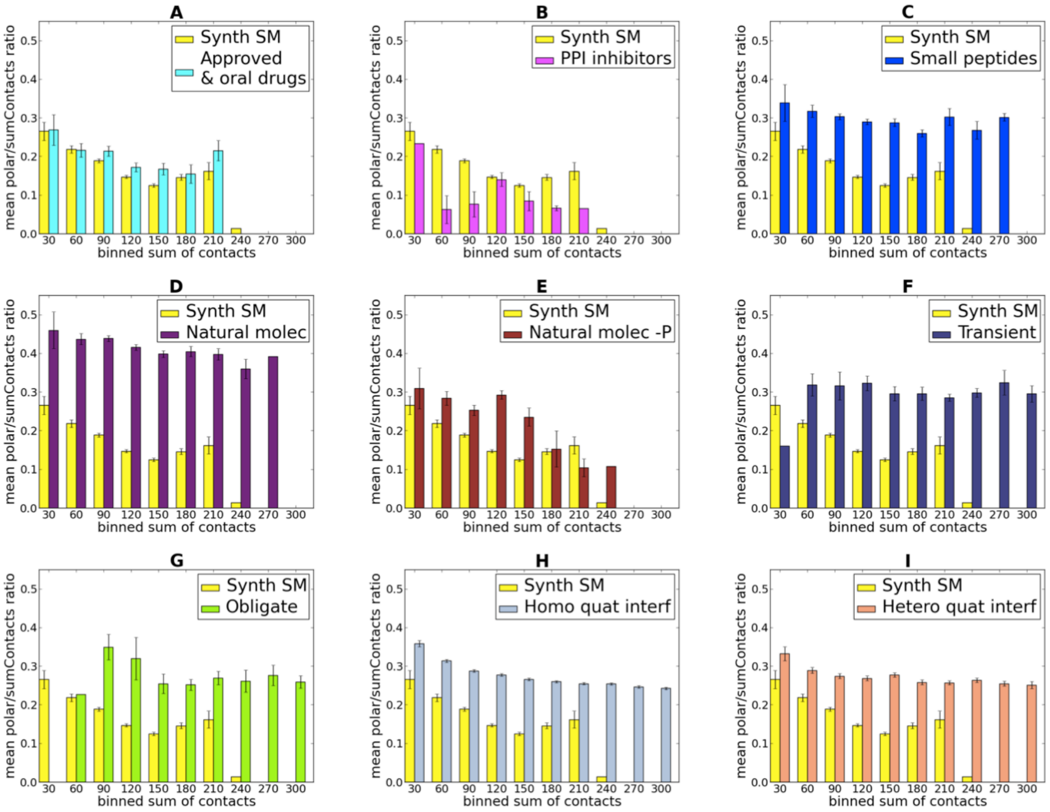

Supplementary Figure SF3.F1. Comparisons of polar/sumContacts ratio means, binned by sum of contacts (polar+apolar), each chart compares synthetic small molecules against the others. (A): synthetic small molecules versus approved and oral drugs. (B): synthetic versus PPI inhibitors. (C): synthetic versus small peptides. (D): synthetic versus natural molecules. (E): synthetic versus natural molecules without phosphor. (F): synthetic versus transient protein-protein dimers. (G): synthetic versus obligate protein-protein dimers. (H): synthetic versus homo quaternary protein-protein interfaces. (I): synthetic versus hetero quaternary protein-protein interfaces. Error bars denote the standard error of the mean.

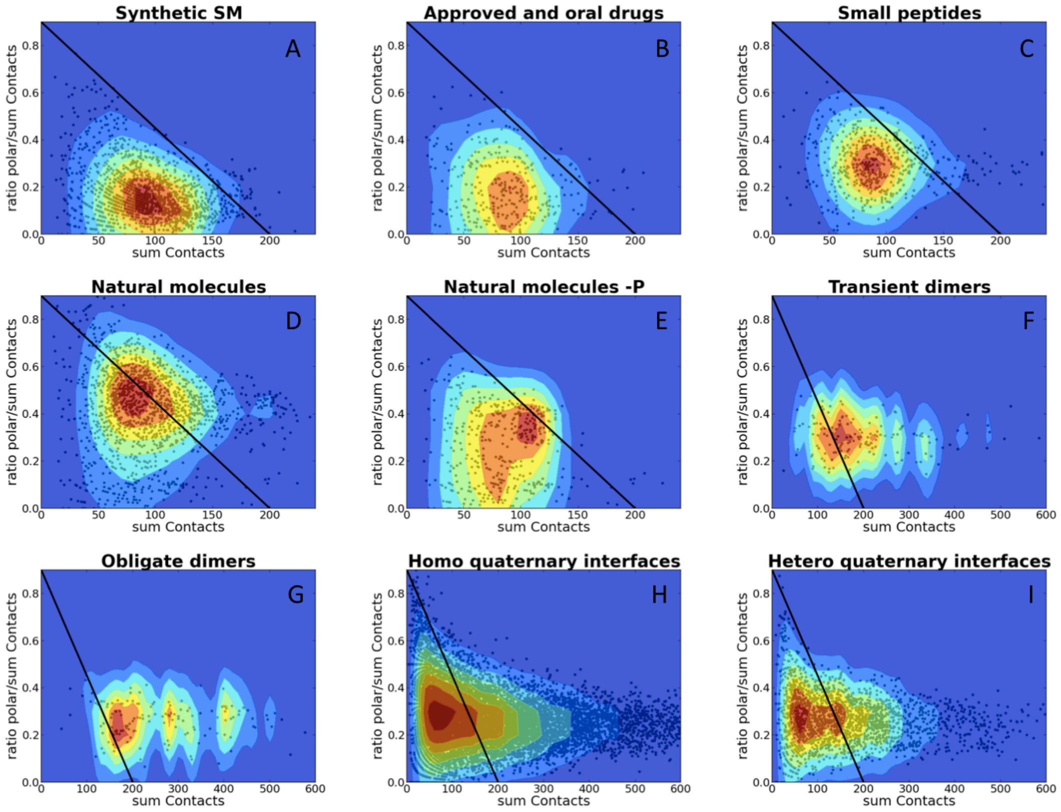

Supplementary Figure SF3.F2. Ratio of polar/(polar+apolar) versus sum of contacts (polar+apolar). Contour levels show the density of points in the graphs, where red denotes high density and pale blue low density. The black line in all the graphs goes between 0.9 ratio to 200 sum of contacts to have the same reference to aid comparison between sets. (A): Synthetic small molecules bound to proteins. (B): Approved and oral drugs bound to proteins. (C): Small peptides bound to proteins. (D): Natural small molecules bound to proteins. (E): Natural small molecules without containing phosphor bound to proteins. (F): Transient protein-protein dimers. (G): Obligate protein-protein dimers. (H): Homo protein-protein interfaces from quaternary structures. (I): Hetero protein-protein interfaces from quaternary structures. For clarity, graphs for protein-protein complexes are plotted up to 600 contacts only.

References

1. Hann MM, Leach AR, Harper G (2001) Molecular Complexity and Its Impact on the Probability of Finding Leads for Drug Discovery. J Chem Inf Comput Sci 41: 856-864.
