# Supplementary material for: What Can We Learn from the Evolution of Protein-Ligand Interactions to Aid the Design of New Therapeutics?
Source: PLoS One. 2012 Dec 11;7(12):e51742. doi: 10.1371/journal.pone.0051742 (PMC3519888; doi:10.1371/journal.pone.0051742)
Supplement: File S4 — Comparison of interaction profiles. (PDF) [file pone.0051742.s006.pdf]

What can we learn from the evolution of protein-ligand interactions to aid the design of new therapeutics?

Alicia P. Higueruelo[1], Adrian Schreyer[1], G. Richard J. Bickerton[1,2], Tom L. Blundell[1] and Will R. Pitt[1,3]

[1]Department of Biochemistry, University of Cambridge, Cambridge, UK

[2]Present address: Division of Biological Chemistry and Drug Discovery, College of Life Sciences, University of Dundee, Dundee, UK

[3]UCB Pharma, Slough, UK

Correspondence should be addressed to APH (alicia@cryst.bioc.cam.ac.uk)

Supplementary File 4

Comparison of interaction profiles

In order to define the statistical significance between the scissors plots of the different subsets, Multiple Linear Regression (MLR) using OLS (Ordinary Least Squares) was used between the apolar regression lines of each set. In addition, the distribution of polar/apolar contact ratio (normalized as polar/[polar+apolar]) between sets was analyzed with non-parametrical tests (Kruskal-Wallis test for comparison of medians), as not all the sets have normal distribution of the contact ratio. This was done to minimize Type I error (concluding there is a significant difference when there is not) due to the heteroscedasticity of the residuals of the regression lines in the scissors plots. Supplementary Table SF4.T1 summarizes the comparisons across all data sets. Supplementary Figure SF4.F1 shows comparison of the distribution of polar/sumContacts ratio for selected subsets.

	App drugs	Oral drugs	PPI Inh	Nat mol	Nat mol -P	Small pep
Synth SM	**-0.03**	0.00	**0.06**	**-0.29**	**-0.14**	**-0.15**
App drugs		0.04	**0.09**	**-0.26**	**-0.11**	**-0.11**
Oral drugs			**0.05**	**-0.30**	**-0.15**	**-0.15**
PPI inh				**-0.35**	**-0.20**	**-0.20**
Nat mol					**0.15**	**0.15**
Nat mol -P						**0.00**

	Obligate	Transient	Homo interf	Hetero interf	PPI inh by SM
Synth SM	**-0.13**	**-0.16**	**-0.12**	**-0.12**	**-0.07**
App drugs	**-0.09**	**-0.12**	**-0.08**	**-0.08**	-0.03
Oral drugs	**-0.13**	**-0.16**	**-0.12**	**-0.12**	-0.07
PPI inh	**-0.18**	**-0.21**	**-0.17**	**-0.17**	**-0.12**
Nat mol	**0.17**	**0.13**	**0.17**	**0.18**	**0.22**
Nat mol –P	0.02	-0.01	0.03	0.03	0.08
Small pep	**0.02**	-0.01	**0.03**	**0.03**	**0.08**
Obligate		**-0.03**	0.01	0.01	0.06
Transient			**0.04**	**0.04**	**0.09**
Homo interf				0.00	0.05
Hetero interf					0.05

Supplementary Table SF4.T1. Differences in medians of the contact ratios (polar/[polar + apolar]) between the different sets of molecules (row - column). Table is divided in two for clarity. Subsets: Synthetic small molecules, App drugs (approved drugs including oral), Oral drugs, PPI inh (protein-protein interactions inhibitors), Nat mol (natural molecules), Nat mol –P (natural molecules that do not contain phosphorous), Small pep (small peptides), Obligate (obligate dimers), Transient (transient dimers), Homo interf (homo quaternary interfaces), Het interf (hetero quaternary interfaces), PPI inh by SM (protein-protein interfaces inhibited by small molecules). Values in bold denote significant differences in medians (P<0.05). Despite the small size of both the PPI SM Inhibitors and PPI SM Inhibited subsets (28 and 15 respectively) they are included for the exceptional insight these cases present.

Values in Supplementary Table SF4.T1 are the differences in medians of the ratio polar/sumContacts for each subset. Synthetic small molecules bound to proteins present on average fewer polar contacts than the other sets, with the exception of the PPI inhibitors that have more apolar contacts. Approved and oral drugs analyzed here present the same interaction profile as synthetic small molecules. Natural molecules comprise the group with most polar contacts on average. When molecules containing phosphorus are removed from the natural molecule set, the average polar contacts decreases by 15%, nevertheless this is the set that engages the most polar interactions. Amongst protein oligomers, homo and hetero interfaces present the same profile for quaternary interfaces, and this is similar to the subset of permanent (obligate) dimers, whereas the transient dimers are slightly (3-4% on average) more polar (in agreement with previous findings [1]) and more similar to the subset of small peptides. Interestingly, the small subset of protein-protein complexes inhibited by small molecules shows a trend that is similar to other protein complexes. However, the small molecules inhibiting them present a more apolar profile than the synthetic small molecules.

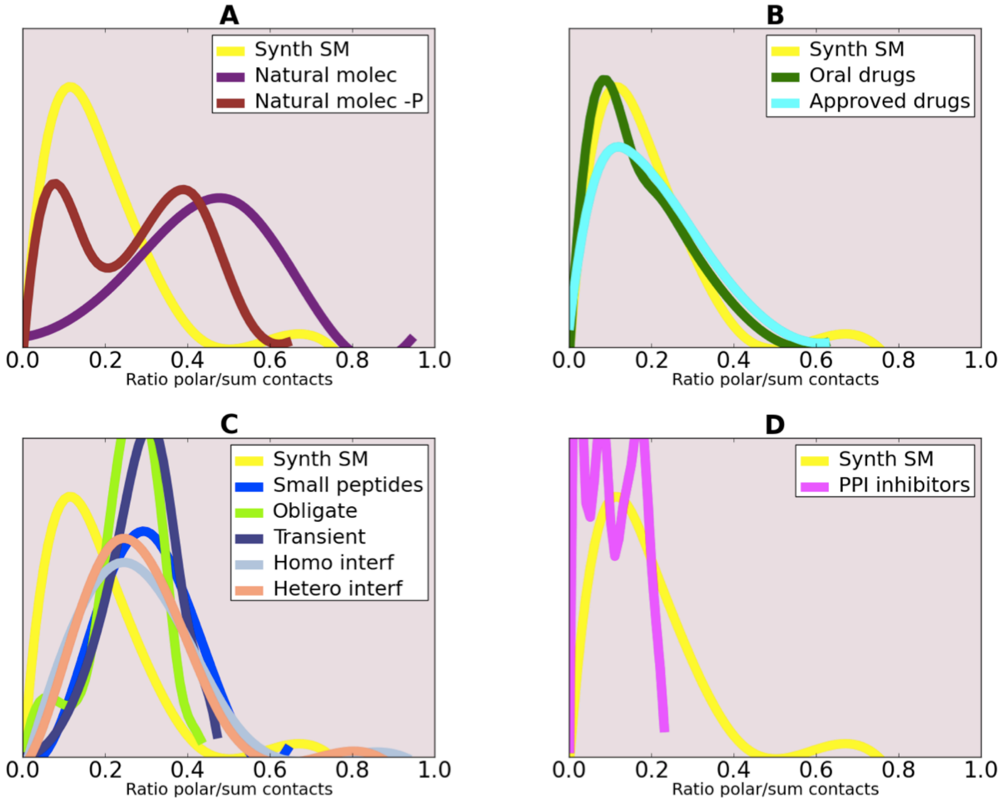

Supplementary Figure SF4.F1. Normalized distributions of the ratio of polar contacts (represented by polar/[polar+apolar]), each chart compares synthetic small molecules against the others. (A): synthetic versus natural small molecules with and without phosphor. (B): synthetic versus approved and oral drugs. (C): synthetic versus small peptides, obligate and transient protein-protein dimers, homo and hetero quaternary protein-protein interfaces. (D): synthetic versus PPI inhibitors.

References

1. Nooren IM, Thornton JM (2003) Diversity of protein-protein interactions. EMBO J 22: 3486-3492.
