# Supplementary material for: What Can We Learn from the Evolution of Protein-Ligand Interactions to Aid the Design of New Therapeutics?
Source: PLoS One. 2012 Dec 11;7(12):e51742. doi: 10.1371/journal.pone.0051742 (PMC3519888; doi:10.1371/journal.pone.0051742)
Supplement: File S5 — Heteroatom content and rotatable bond count. (PDF) [file pone.0051742.s007.pdf]

What can we learn from the evolution of protein-ligand interactions to aid the design of new therapeutics?

Alicia P. Higueruelo[1], Adrian Schreyer[1], G. Richard J. Bickerton[1,2], Tom L. Blundell[1] and Will R. Pitt[1,3]

[1]Department of Biochemistry, University of Cambridge, Cambridge, UK

[2]Present address: Division of Biological Chemistry and Drug Discovery, College of Life Sciences, University of Dundee, Dundee, UK

[3]UCB Pharma, Slough, UK

Correspondence should be addressed to APH (alicia@cryst.bioc.cam.ac.uk)

Supplementary File 5

Heteroatom content and rotatable bond count

Supplementary Table SF5.T1 and Supplementary Figure SF5.F1 summarize the comparisons between the ratios of heteroatoms and rotatable bonds by heavy atoms for the small molecule subsets. Only distinct molecules are considered for these calculations. The number of molecules in each set is as follows: synthetic small molecules (1,206), approved drugs (95), oral drugs (68), protein-protein interactions inhibitors (25), natural molecules (283), natural molecules that do not contain phosphor (179) and small peptides (467).

**Het/N_at**	**App drugs**	**Oral drugs**	**PPI inh**	**Nat mol**	**Nat mol -P**	**Small pep**
Synth SM	-0.01	-0.02	0.01	**-0.19**	0.02	**-0.10**
App drugs		-0.01	0.02	**-0.18**	0.03	**-0.09**
Oral drugs			0.04	**-0.16**	0.04	**-0.08**
PPI inh				**-0.20**	0.00	**-0.11**
Nat mol					**0.20**	**0.09**
Nat mol -P						**-0.12**

**Rot/N_at**	**App drugs**	**Oral drugs**	**PPI inh**	**Nat mol**	**Nat mol -P**	**Small pep**
Synth SM	-0.01	-0.01	**-0.07**	**-0.04**	0.00	**-0.24**
App drugs		0.00	**-0.07**	**-0.03**	0.01	**-0.24**
Oral drugs			**-0.07**	**-0.03**	0.01	**-0.24**
PPI inh				0.03	**0.08**	**-0.17**
Nat mol					**0.04**	**-0.20**
Nat mol -P						**-0.25**

Supplementary Table SF5.T1. Difference in medians of the ratios (number of heteroatoms/number of heavy atoms, upper table) and (number of rotatable bonds/number of heavy atoms, lower table). Differences are between the different set of small molecules (row – column). Subsets: Synthetic small molecules, App drugs (approved drugs including oral), Oral drugs, PPI inh (protein-protein interactions inhibitors), Nat mol (natural molecules), Nat mol –P (natural molecules that do not contain phosphor), Small pep (small peptides). Values in bold denote significant differences in medians (P<0.05).

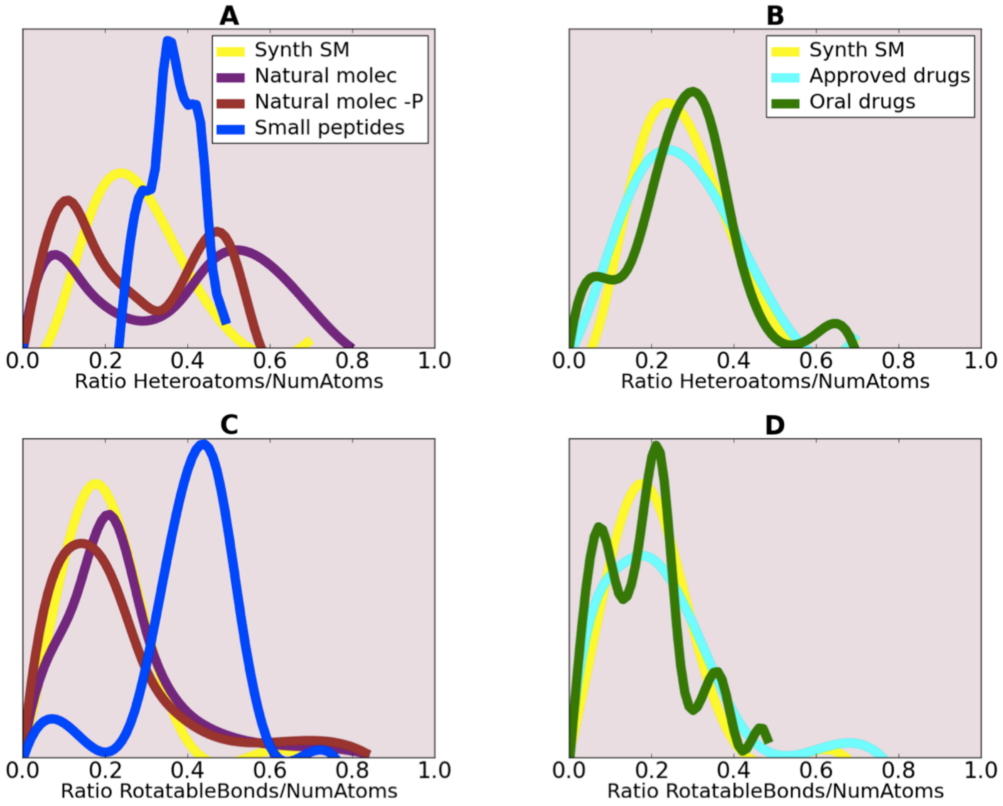

Supplementary Figure SF5.F1. Normalized distributions of the ratio of heteroatoms and rotatable bonds by number of heavy atoms. (A): Distribution of the ratio of number of heteroatoms by number of heavy atoms for synthetic small molecules, natural molecules, natural molecules without phosphor and small peptides. (B): Distribution of the ratio number of heteroatoms versus number of heavy atoms for synthetic small molecules, approved and oral drugs. (C): Distribution of the ratio of number of rotatable bonds by number of heavy atoms for synthetic small molecules, natural molecules, natural molecules without phosphor and small peptides. (D): Distribution of the ratio of number of rotatable bonds by number of heavy atoms for synthetic small molecules, approved and oral drugs.
