# Supplementary material for: What Can We Learn from the Evolution of Protein-Ligand Interactions to Aid the Design of New Therapeutics?
Source: PLoS One. 2012 Dec 11;7(12):e51742. doi: 10.1371/journal.pone.0051742 (PMC3519888; doi:10.1371/journal.pone.0051742)
Supplement: File S6 — Free energy of ligand binding versus the polar ratio of contacts [polar/(polar+apolar)] for the synthetic small molecules set and the small peptide set. (PDF) [file pone.0051742.s008.pdf]

# What can we learn from the evolution of protein-ligand interactions to aid the design of new therapeutics?

Alicia P. Higueruelo[1], Adrian Schreyer[1], G. Richard J. Bickerton[1,2], Tom L. Blundell[1] and Will R. Pitt[1,3]

[1]Department of Biochemistry, University of Cambridge, Cambridge, UK

[2]Present address: Division of Biological Chemistry and Drug Discovery, College of Life Sciences, University of Dundee, Dundee, UK

[3]UCB Pharma, Slough, UK

Correspondence should be addressed to APH (alicia@cryst.bioc.cam.ac.uk)

## Supplementary File 6

From the 1,206 distinct small molecules in the synthetic small molecules set, almost 700 have affinity data (Kd, Ki or IC50) which can be obtained from the implementation of PDBBind [1] in CREDO. Unfortunately, not many natural molecules have affinity data, and comparison with how these molecules achieve high potency cannot be achieved with the current data available. However, there are 112 distinct small peptides with Kd, Ki or IC50 in CREDO. Free energy of ligand binding (Kcal/mol) for qualitative comparison was derived using: $\Delta G = -RT \ln Kd$, where R is the gas constant, T is the temperature in Kelvin (taken as 300K, ambient temperature) and Kd is the equilibrium dissociation constant of the binding event. When Kd was not available, IC50 or Ki were taken instead. Supplementary Figure SF6.F1 shows there is no relation between the binding energy and the proportion of polar contacts made by the small molecules or small peptides with their protein partners. Higher polar ratios occur only for synthetic weak binders with molecular weights below 300Da. As seen before, for the synthetic

small molecules, only weakly binding, small fragments can achieve a high proportion of polar interactions. This is not the case for small peptides, where high polar contact ratio can be achieved across a wide range of affinities.

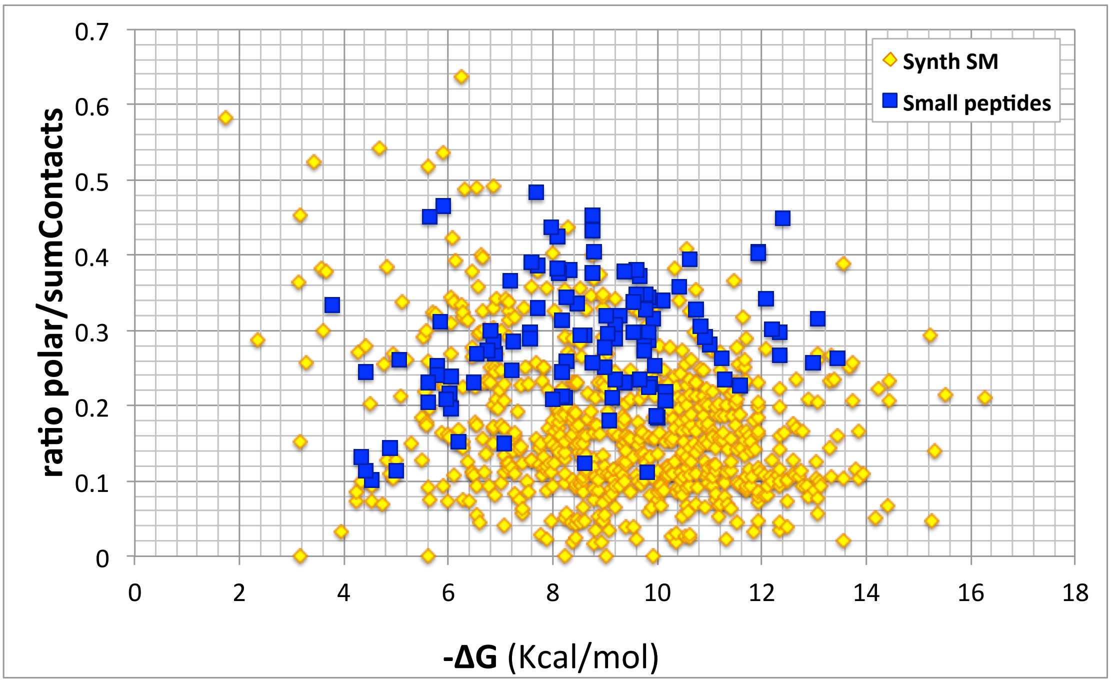


Supplementary Figure SF6.F1. Free energy of ligand binding versus the polar ratio of contacts [polar/(polar+apolar)] for the synthetic small molecules set (yellow) and the small peptide set (blue).

## References

1. Wang R, Fang X, Lu Y, Wang S (2004) The PDBbind database: collection of binding affinities for protein-ligand complexes with known three-dimensional structures. J Med Chem 47: 2977-2980.
