# Supplementary material for: What Can We Learn from the Evolution of Protein-Ligand Interactions to Aid the Design of New Therapeutics?
Source: PLoS One. 2012 Dec 11;7(12):e51742. doi: 10.1371/journal.pone.0051742 (PMC3519888; doi:10.1371/journal.pone.0051742)
Supplement: File S7 — Free energy of ligand binding versus the number of atoms of the ligand for the synthetic small molecules set and the small peptide set. (PDF) [file pone.0051742.s009.pdf]

What can we learn from the evolution of protein-ligand interactions to aid the design of new therapeutics?

Alicia P. Higueruelo[1], Adrian Schreyer[1], G. Richard J. Bickerton[1,2], Tom L. Blundell[1] and Will R. Pitt[1,3]

[1]Department of Biochemistry, University of Cambridge, Cambridge, UK

[2]Present address: Division of Biological Chemistry and Drug Discovery, College of Life Sciences, University of Dundee, Dundee, UK

[3]UCB Pharma, Slough, UK

Correspondence should be addressed to APH (alicia@cryst.bioc.cam.ac.uk)

Supplementary File 7

Analysis of the free energy of binding versus the number of atoms for synthetic small molecule and small peptide subsets (Supplementary Figure SF7.F1) confirms that there is no correlation between the number of atoms and the free energy of binding for small peptides. However, they are less efficient than synthetic small molecules as they use more atoms to achieve the same affinity. Furthermore, the values for binding affinities are confined in the range of what can be measured (tens of milimolar that translates into ~2Kcal/mol to picomolar than translates into ~16Kcal/mol). In this way, peptides are able to sample binding energies between 4Kcal/mol to 14Kcal/mol regardless of their size.

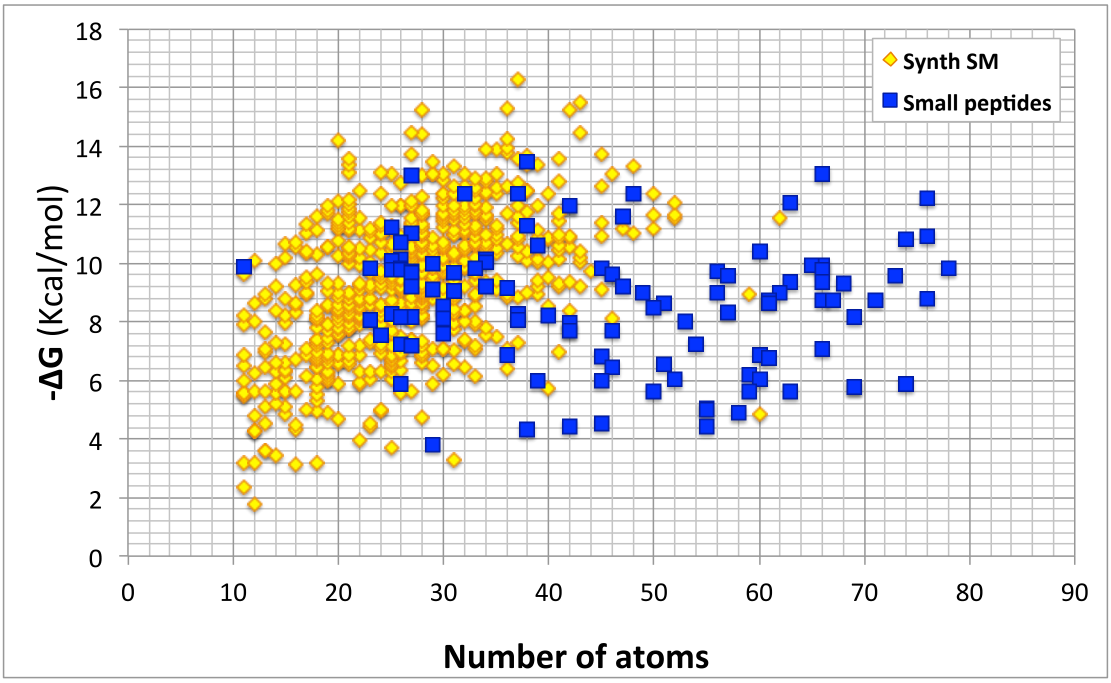

Supplementary Figure SF7.F1. Free energy of ligand binding versus the number of atoms of the ligand. Synthetic small molecule set is plotted in yellow, and small peptide set in blue.

In Supplementary Figure SF7.F1 there are three synthetic molecules with circa 60 atoms. The most potent is a symmetric cyclic urea HIV-1 protease inhibitor with 4nM affinity (PDB 1BWB). The weakest, with 300uM measured affinity is the deoxy-bigchaps bound to IGF-1 through the steroid-like head, the two polar tails of the molecule are floating in the solvent (PDB 1IMX). The third molecule binds to calmodium with an affinity of 3uM; this is a large and complex non-planar molecule, which binds to residues from the N- and C-terminal domains of calmodulin and induces a major conformational change (PDB 1XA5).
