# Supplementary material for: What Can We Learn from the Evolution of Protein-Ligand Interactions to Aid the Design of New Therapeutics?
Source: PLoS One. 2012 Dec 11;7(12):e51742. doi: 10.1371/journal.pone.0051742 (PMC3519888; doi:10.1371/journal.pone.0051742)
Supplement: File S8 — Protein-protein complexes inhibited by small molecules. (PDF) [file pone.0051742.s010.pdf]

What can we learn from the evolution of protein-ligand interactions to aid the design of new therapeutics?

Alicia P. Higueruelo[1], Adrian Schreyer[1], G. Richard J. Bickerton[1,2], Tom L. Blundell[1] and Will R. Pitt[1,3]

[1]Department of Biochemistry, University of Cambridge, Cambridge, UK

[2]Present address: Division of Biological Chemistry and Drug Discovery, College of Life Sciences, University of Dundee, Dundee, UK

[3]UCB Pharma, Slough, UK

Correspondence should be addressed to APH (alicia@cryst.bioc.cam.ac.uk)

Supplementary File 8

Protein-protein complexes inhibited by small molecules

Regarding protein-protein complexes inhibited by small molecules, Supplementary Figure SF8.F1 shows the comparison of the residue propensities for the protein interfaces where structures have been independently structurally determined for complexes with both protein and small molecule inhibitor. These cases are S100B, IL-2, MDM2, ZipA, XIAP, Bcl-XL, Bcl-2, and TNF alpha. There are nine distinct UniProt protein-small molecule complexes and seven protein-protein complexes. The high standard error bars denote the variability and the small size of the sets. However, it is clear from this comparison that the small molecules avoid contact with the available charged and polar residues in favor of interacting with the hydrophobic ones.

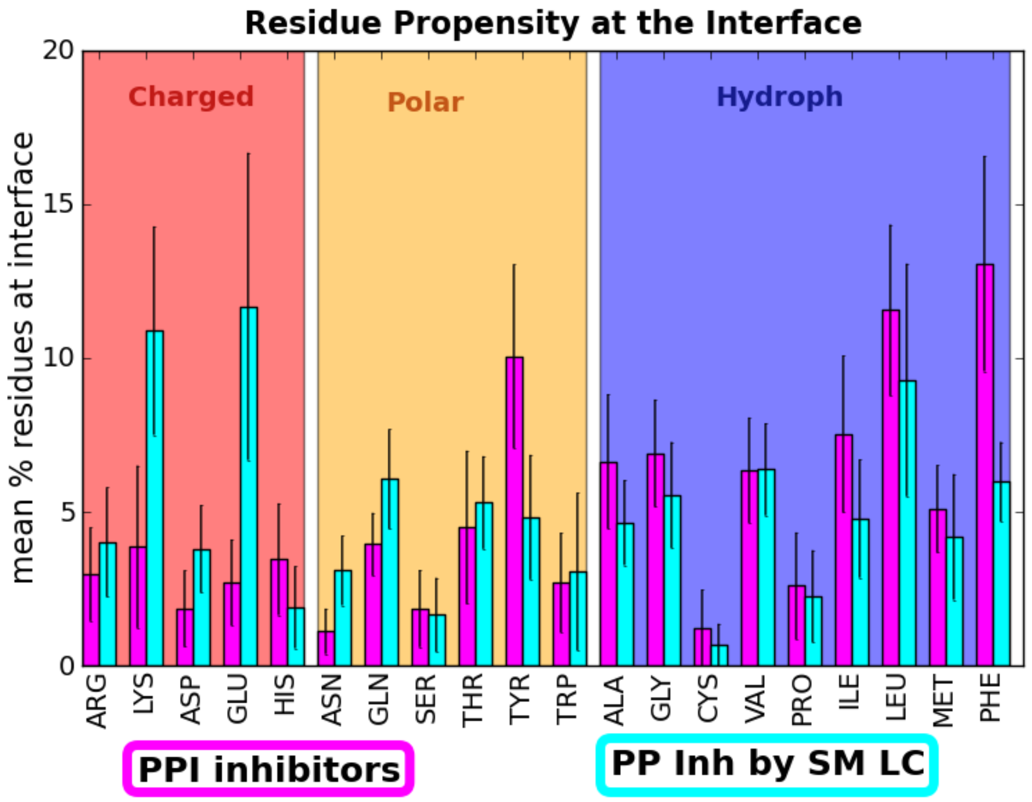

Supplementary Figure SF8.F1. Comparisons of residue propensities at the binding sites for small molecule protein-protein inhibitors (magenta) versus protein-protein complexes inhibited by them (cyan), note these subsets are small (9 and 7 complexes respectively). For protein-protein complexes only the long chain (LC) is considered. Bar heights represent the mean percentage of each residue at the interface. Error bars denote the standard error of the mean. The background color represents whether the residue is charged (red), polar (orange) or hydrophobic (blue).

Indeed, small molecules occupy only a portion of the protein-protein binding interface, and they tend to maximize the hydrophobic contacts rather than the polar ones. This may be a result of the small molecules binding at the hot spots of the interfaces, especially in the standard medicinal chemistry settings where the pursuit of affinity is prioritized. However, these molecules seem to be missing the specific contacts that would confer them selectivity towards these interfaces. However, hydrogen bond matching at an open interface might require a degree of flexibility that it is harder to design and successfully achieve, and it could explain the low content of hydrogen bonds in the first successful small molecule

inhibitors of protein-protein interactions. Supplementary Figures SF8.F2 to SF8.F8 show a graphical representation of these binding modes.

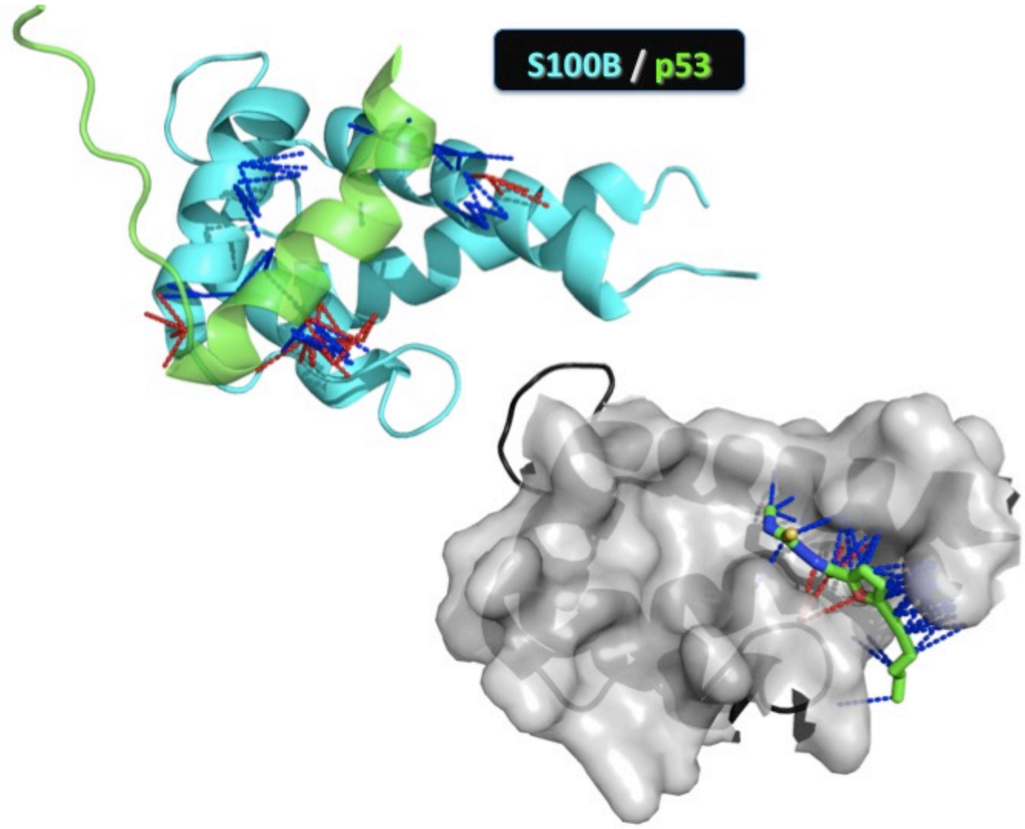

Supplementary Figure SF8.F2. **S100B**. Upper left: 1DT7, S100B (cyan) with the C-terminal negative regulatory domain of p53 (green). Ratio [polar/(polar+apolar)] is 0.34. Lower right: 3GK1, S100B (dark grey) with small molecule inhibitor (green). Ratio [polar/(polar+apolar)] is 0.12. The surface covers the S100B residues that are within 4.5Å of p53. For both complexes polar contacts are red dotted lines and apolar are blue dotted lines.

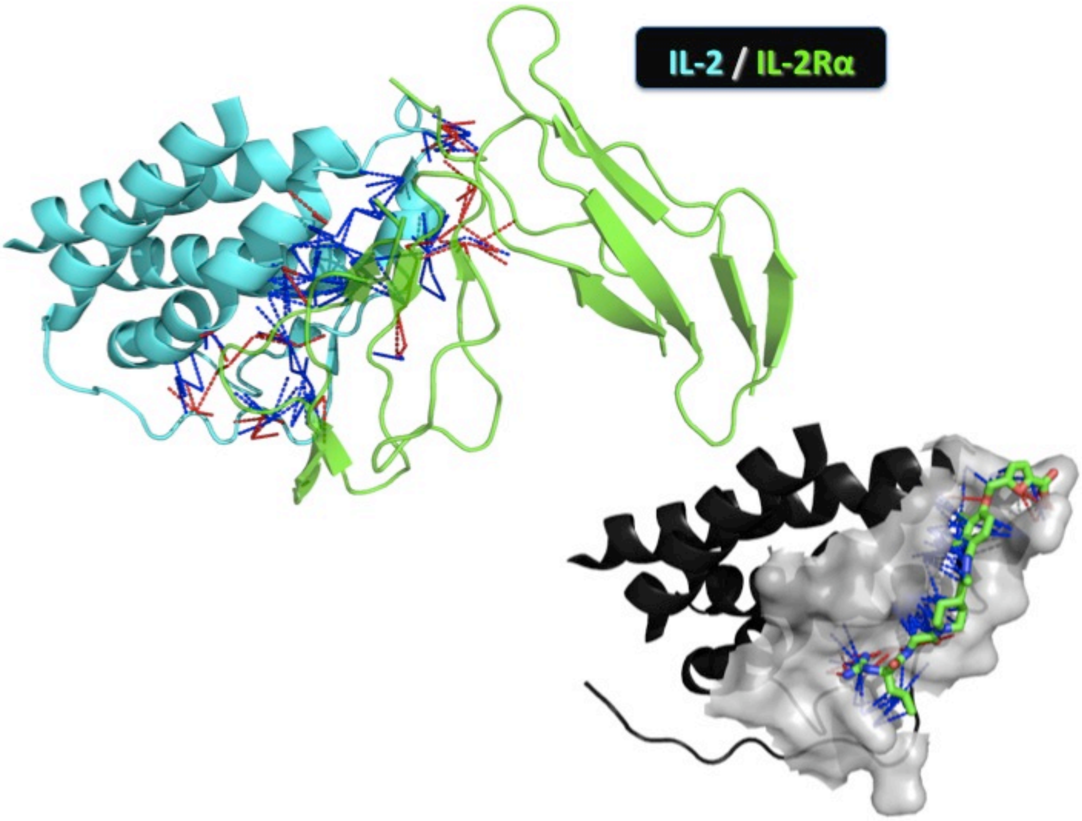

Supplementary Figure SF8.F3. **IL-2**. Upper left: 1Z92, IL-2 (cyan) bound to IL-2R alpha subunit (green). Ratio [polar/(polar+apolar)] is 0.35. Lower right: 1PY2, IL-2 (dark grey) with a Sunesis small molecule inhibitor (green). Ratio [polar/(polar+apolar)] is 0.21. The surface covers the IL-2 residues that are within 4.5Å of the IL-2Ra. For both complexes polar contacts are red dotted lines and apolar are blue dotted lines.

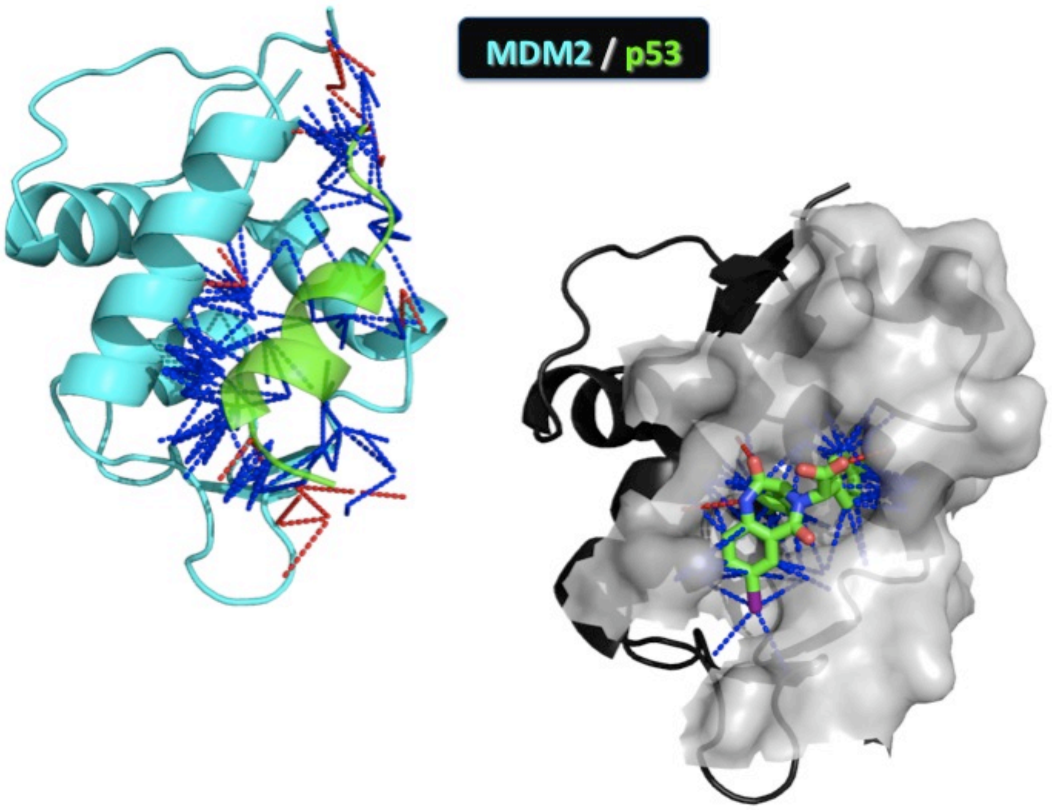

Supplementary Figure SF8.F4. **MDM2**. Upper left: 1YCR, MDM2 (cyan) bound to the transactivation domain of p53 (green). Ratio [polar/(polar+apolar)] is 0.14. Lower right: 1T4E, MDM2 (dark grey) with a benzodiazepine inhibitor (green). Ratio [polar/(polar+apolar)] is 0.03. The surface covers the MDM2 residues that are within 4.5Å of the p53. For both complexes polar contacts are red dotted lines and apolar are blue dotted lines.

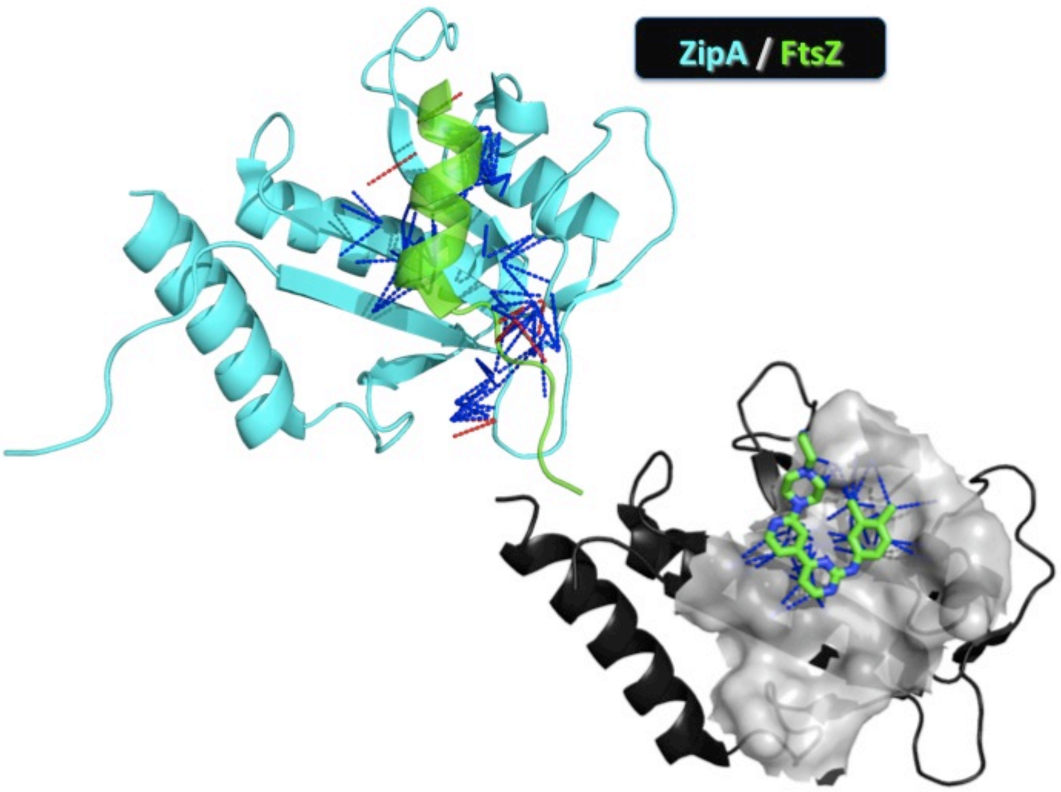

Supplementary Figure SF8.F5. **ZipA**. Upper left: 1F47: ZipA (cyan) bound to a fragment of FtsZ (green). Ratio [polar/(polar+apolar)] is 0.10. Lower right: 1Y2F: ZipA (dark grey) with an aminopyrimidine inhibitor (green). The surface covers the ZipA residues that within 4.5Å of the FtsZ. For both complexes polar contacts are red dotted lines and apolar are blue dotted lines. Note the small molecule does not engage a single polar contact.

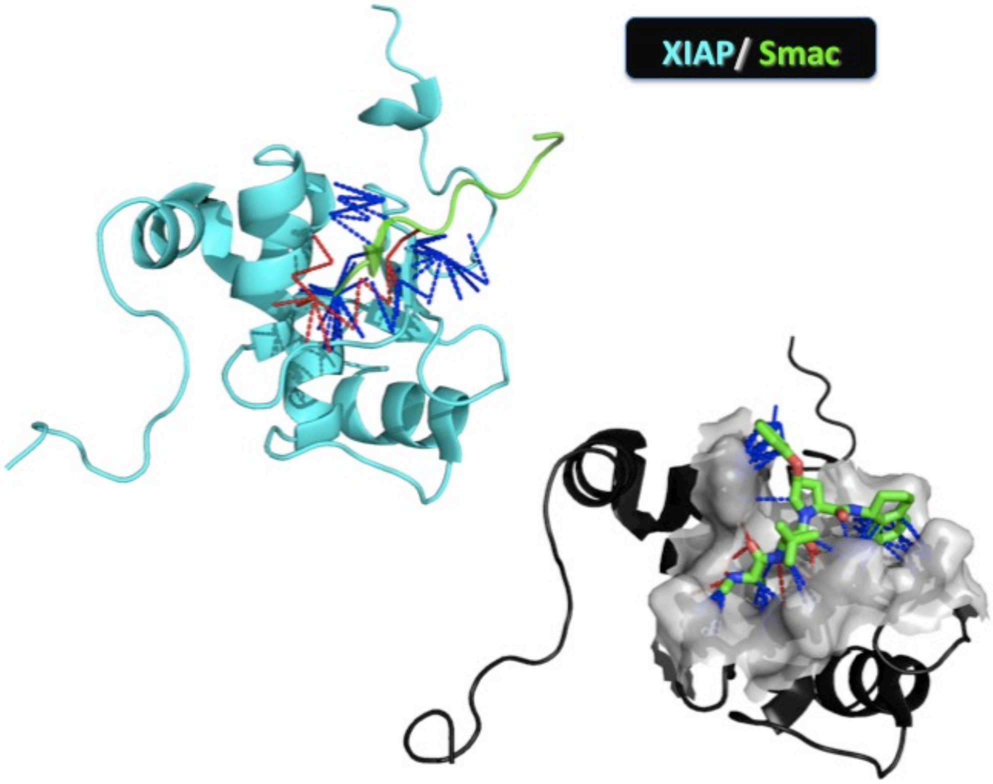

Supplementary Figure SF8.F6. **XIAP**. Upper left: 1G3F, BIR3 domain of XIAP (cyan) bound to an active nine-residue peptide derived from Smac (green). Ratio [polar/(polar+apolar)] is 0.22. Lower right: 1TFT, XIAP (dark grey) with a small molecule inhibitor (green). Ratio [polar/(polar+apolar)] is 0.12. The surface covers the XIAP residues that within 4.5Å of the Smac fragment. For both complexes polar contacts are red dotted lines and apolar are blue dotted lines.

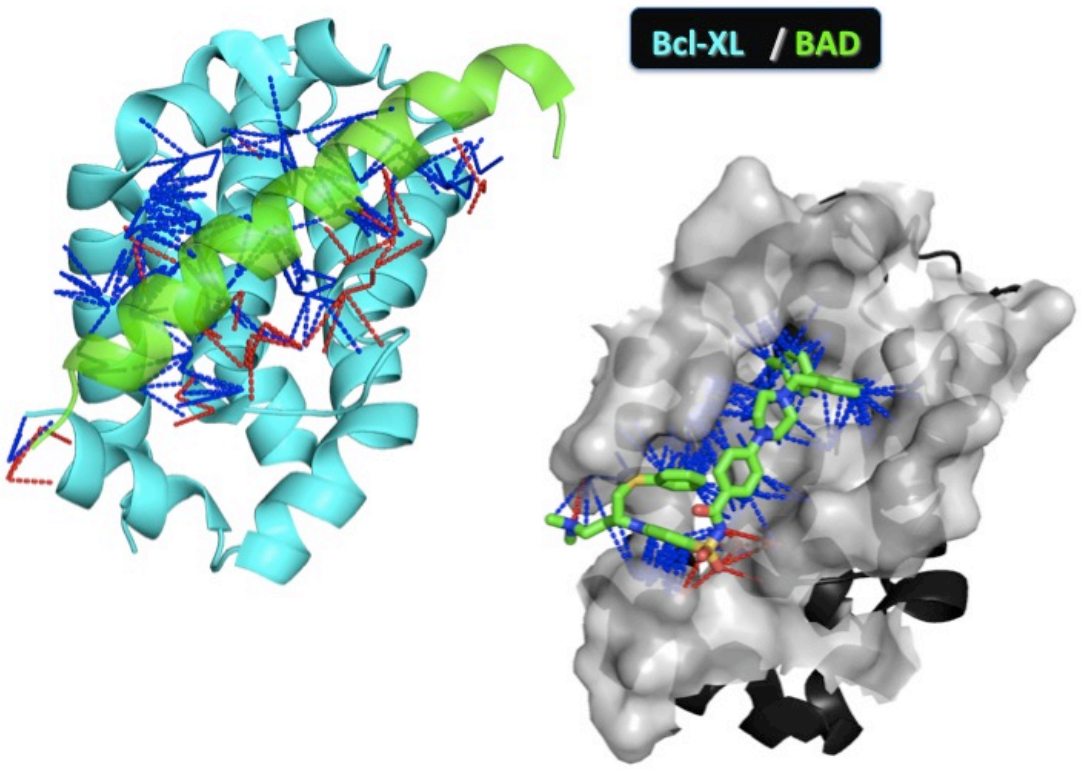

Supplementary Figure SF8.F7. **Bcl-XL**. Upper left: 2BZW, Bcl-XL (cyan) bound to BAD (green). Ratio [polar/(polar+apolar)] is 0.19. Lower right: 2YXJ, Bcl-XL (dark grey) with the Abbott compound ABT-737 (green). Ratio [polar/(polar+apolar)] is 0.08. The surface covers the Bcl-XL residues that within 4.5Å of BAD. For both complexes polar contacts are red dotted lines and apolar are blue dotted lines. Note that the small molecule only engages polar contacts at the bottom of the picture and it is bound to Bcl-XL mainly though apolar contacts.

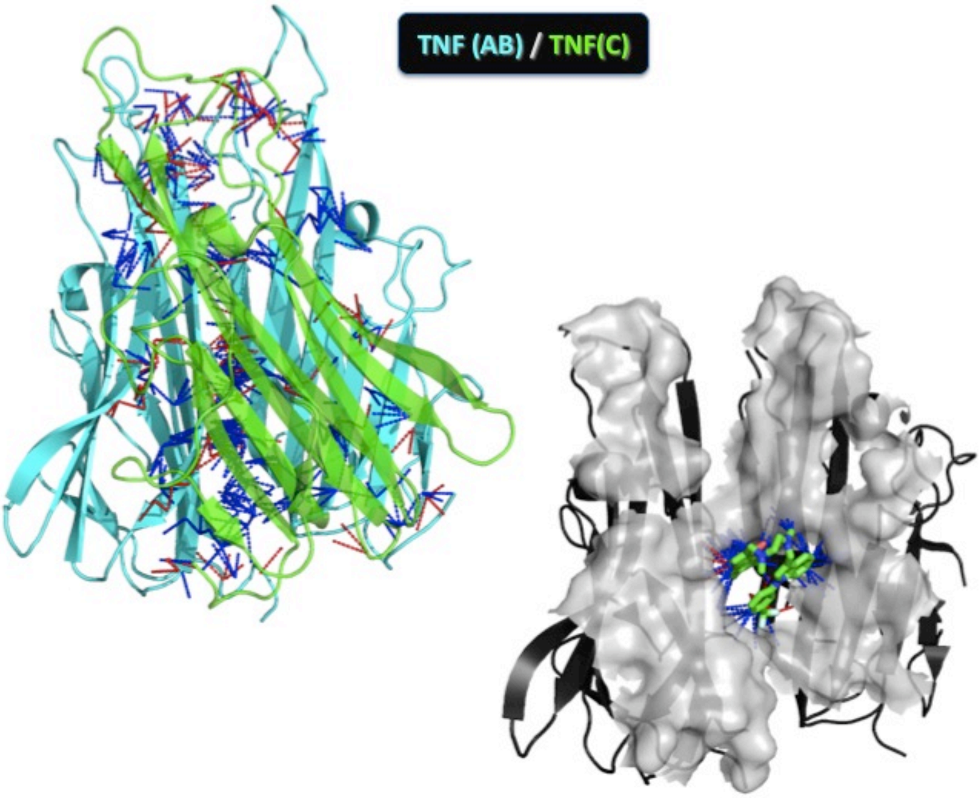

Supplementary Figure SF8.F8. **TNF**. Upper left: 1TNF, TNF alpha trimer, two chains are colored in cyan and the third in green. Ratio [polar/(polar+apolar)] is 0.30. Lower right: 2AZ5, two chains of the TNF trimer (dark grey) bound to a small molecule (green) that accelerates subunit dissociation. Ratio [polar/(polar+apolar)] is 0.12. The surface covers the residues in these chains that are within 4.5Å of the third chain. For both complexes polar contacts are red dotted lines and apolar are blue dotted lines. Note small molecule binds to an area where there are no interactions in the trimer.
