# Supplementary material for: What Can We Learn from the Evolution of Protein-Ligand Interactions to Aid the Design of New Therapeutics?
Source: PLoS One. 2012 Dec 11;7(12):e51742. doi: 10.1371/journal.pone.0051742 (PMC3519888; doi:10.1371/journal.pone.0051742)
Supplement: Table S1 — Approved and oral small molecule drugs that engage more than 40% polar contacts with their bound protein. (PDF) [file pone.0051742.s011.pdf]

	HetID (PDB)	Ratio	alogP	Drug type	DrugBank ID
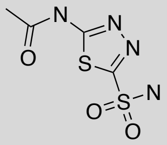	AZM (3HS4)	0.63	-1.3	Approved oral	DB00819 Acetazolamide
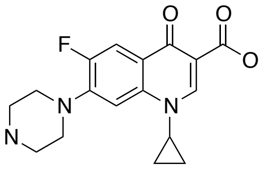	CPF (1T9U)	0.61	-1.3	Approved oral	DB00537 Ciprofloxacin
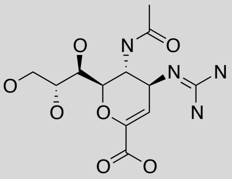	ZMR (2HTQ)	0.60	-5.0	Approved inhalation	DB00558 Zanamivir
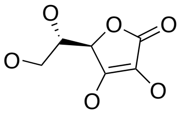	ASC (1F9G)	0.58	-1.9	Approved nutraceutical oral	DB00126 Ascorbic acid (vitamin C)
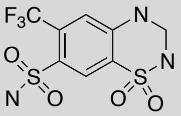	HFZ (3ILU)	0.53	0.0	Approved oral	DB00774 Hydro-flumethiazide
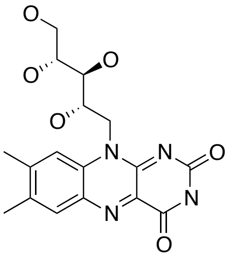	RBF (3DDY)	0.52	0.1	Approved nutraceutical oral	DB00140 Riboflavin (vitamin B2)
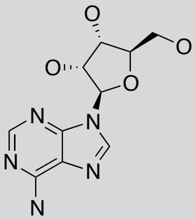	AND (1UAY)	0.48	-1.9	Approved intravenous	DB00640 Adenosine
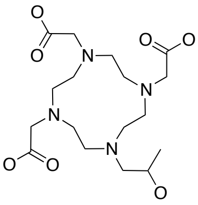	DO3 (2QMI)	0.48	-8.9	Approved intravenous	DB00597 Gadoteridol

	HetID (PDB)	Ratio	alogP	Drug type	DrugBank ID
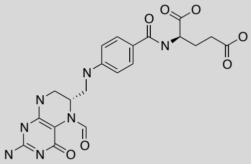	FON (3GEH)	0.46	-5.0	Experimental Vitamin B complex	DB03256 Folinic acid
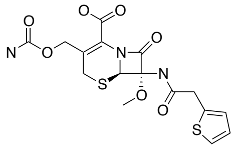	CFX (1I2W)	0.46	-1.6	Approved antibiotic intravenous	DB01331 Cefoxitin
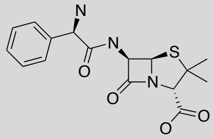	AIC (2RDD)	0.46	-2.3	Approved oral antibiotic	DB00415 Ampicillin
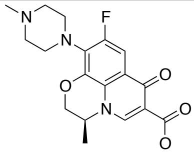	LFX (3K9F)	0.44	-1.4	Approved oral antibacterial	DB01137 Levofloxacin
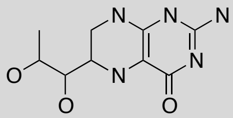	H4B (2DTT)	0.43	-1.1	Approved nutraceutical oral	DB00360 Tetrahydro-biopterin
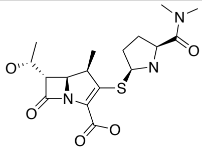	MER (1H8Y)	0.42	-4.9	Approved intravenous antibiotic	DB00760 Meropenem
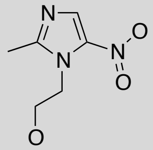	2MN (1W3R)	0.41	-0.2	Approved oral	DB00916 Metronidazole
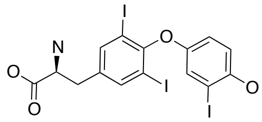	T3 (2PIV)	0.41	1.5	Approved oral	DB00279 Liothyronine
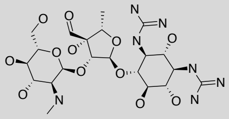	SRY (3HAV)	0.40	-7.7	Approved intramuscular antibiotic	DB01082 Streptomycin
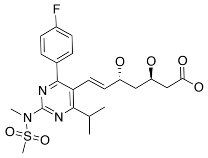	FBI (1HWL)	0.40	0.9	Approved oral	DB01098 Rosuvastatin

Supplementary Table S1. Approved and oral small molecule drugs that engage more than 40% polar contacts with their bound protein. HetID is the residue identifier for the ligand in the PDB. Ratio refers to the ratio of polar contacts as [polar/(polar+apolar)]. In the chemical structure representation, all hydrogens are omitted.
