# Supplementary material for: What Can We Learn from the Evolution of Protein-Ligand Interactions to Aid the Design of New Therapeutics?
Source: PLoS One. 2012 Dec 11;7(12):e51742. doi: 10.1371/journal.pone.0051742 (PMC3519888; doi:10.1371/journal.pone.0051742)
Supplement: Table S2 — Number of entries in each set of molecules. (PDF) [file pone.0051742.s012.pdf]

Set	Unique by Complex	Unique UniProt	Unique SCOP families
**Synthetic small molecules**	1,525 (1,206)	518 (385)	165 (143)
**Approved drugs**	201 (95)	155 (76)	67 (46)
**Oral drugs**	134 (68)	93 (49)	24 (19)
**Protein-protein interaction inhibitors**	30 (25)	9 (9)	7 (7)
**Natural molecules**	1505 (283)	1159 (216)	346 (134)
**Small peptides**	557 (467)	288 (238)	98 (83)
**Obligate dimers**	161	161	293
**Transient dimers**	154	154	183
**Homo quaternary interfaces**	12,034	7,177	2,711
**Hetero quaternary interfaces**	2,271	1,709	897
**Protein-protein complexes SM inhibited**	15	15	13

Supplementary Table S2. Number of entries in each set of molecules. The non-redundant sets are considering non-redundant set of interactions for the complexes (protein-ligand or protein-protein interaction). From these sets protein redundancy is removed by selecting unique UniProt identifiers and removed structural domains redundancy by selecting unique SCOP families. Numbers in parenthesis are the number of unique small molecules in each set. Numbers for unique UniProt and SCOP families for protein complexes refer to distinct pairs of UniProt identifiers or SCOP family respectively.
